# Coral microbiome composition along the northern Red Sea suggests high plasticity of bacterial and specificity of endosymbiotic dinoflagellate communities

**DOI:** 10.1186/s40168-019-0776-5

**Published:** 2020-02-06

**Authors:** Eslam O. Osman, David J. Suggett, Christian R. Voolstra, D. Tye Pettay, Dave R. Clark, Claudia Pogoreutz, Eugenia M. Sampayo, Mark E. Warner, David J. Smith

**Affiliations:** 1 Coral Reef Research Unit, School of Life Sciences, University of Essex, Colchester, CO4 3SQ UK; 2 Marine Biology Department, Faculty of Science, Al-Azhar University, Nasr City, Cairo 11448 Egypt; 3 Climate Change Cluster, University of Technology Sydney, Sydney, New South Wales 2007 Australia; 4 Red Sea Research Center, Division of Biological and Environmental Science and Engineering (BESE), King Abdullah University of Science and Technology (KAUST), Thuwal, Saudi Arabia; 5 Department of Biology, University of Konstanz, 78457 Konstanz, Germany; 6 School of Marine Science and Policy, College of Earth, Ocean, and Environment, University of Delaware, Lewes, DE 19958 USA; 7 ARC Centre of Excellence for Coral Reef Studies, School of Biological Sciences, The University of Queensland, St. Lucia, 4072 QLD Australia

**Keywords:** Microbial community, 16S rRNA gene profiling, *Symbiodiniaceae*, Coral acclimatization, Holobiont, Climate change, Future Oceans

## Abstract

**Background:**

The capacity of reef-building corals to tolerate (or adapt to) heat stress is a key factor determining their resilience to future climate change. Changes in coral microbiome composition (particularly for microalgal endosymbionts and bacteria) is a potential mechanism that may assist corals to thrive in warm waters. The northern Red Sea experiences extreme temperatures anomalies, yet corals in this area rarely bleach suggesting possible refugia to climate change. However, the coral microbiome composition, and how it relates to the capacity to thrive in warm waters in this region, is entirely unknown.

**Results:**

We investigated microbiomes for six coral species (*Porites nodifera, Favia favus, Pocillopora damicornis*, *Seriatopora hystrix*, *Xenia umbellata*, and *Sarcophyton trocheliophorum*) from five sites in the northern Red Sea spanning 4° of latitude and summer mean temperature ranges from 26.6 °C to 29.3 °C. A total of 19 distinct dinoflagellate endosymbionts were identified as belonging to three genera in the family *Symbiodiniaceae* (*Symbiodinium*, *Cladocopium*, and *Durusdinium*). Of these, 86% belonged to the genus *Cladocopium*, with notably five novel types (19%). The endosymbiont community showed a high degree of host-specificity despite the latitudinal gradient. In contrast, the diversity and composition of bacterial communities of the surface mucus layer (SML)—a compartment particularly sensitive to environmental change—varied significantly between sites, however for any given coral was species-specific.

**Conclusion:**

The conserved endosymbiotic community suggests high physiological plasticity to support holobiont productivity across the different latitudinal regimes. Further, the presence of five novel algal endosymbionts suggests selection of certain genotypes (or genetic adaptation) within the semi-isolated Red Sea. In contrast, the dynamic composition of bacteria associated with the SML across sites may contribute to holobiont function and broaden the ecological niche. In doing so, SML bacterial communities may aid holobiont local acclimatization (or adaptation) by readily responding to changes in the host environment. Our study provides novel insight about the selective and endemic nature of coral microbiomes along the northern Red Sea refugia.

## Introduction

Coral reefs have dramatically declined during the last two decades through the mortality of reef-building species driven by frequent and intense heatwaves [1, 2]. Efforts to predict if and how corals will survive into the future has resulted in intensive research to understand coral thermal tolerance across environments [3] and through time [4]. Corals can persist in relatively extreme habitats such as shallow pools [5], reef flats [6], and mangroves [7, 8], or marginally “hot” reef systems such as those within parts of the Persian-Arabian Gulf [9] and the Red Sea [4]. Therefore, coral populations that already exist at high ambient water temperatures have become important model systems to evaluate the different mechanisms with which thermal tolerance may be acquired [10–12].

Coral thermal tolerance is ultimately determined by the genetic composition of the holobiont (i.e., the coral host and its associated microbiome: endosymbiotic dinoflagellates, bacteria, virus, fungi, archaea, and endolithic algae—sensu Rohwer et al. [13]). Some corals exhibit a broad capacity to adapt to different thermal histories by frontloading genes that promote heat stress tolerance [3, 14], and/or potentially through shifting their microbial community [15–17]. Endosymbiont genotypes or species (family *Symbiodiniaceae* [18]) associated with corals play an important role in the adaptation of corals living under extreme environments [19, 20]. Endosymbiont response to environmental fluctuations varies greatly between (and within) species/genotypes [21], and the persistence of certain genotypes can influence coral stress tolerance [22]. A new species, *Cladocopium thermophilum*, resides in extreme warm waters (> 35 °C) of the Persian-Arabian Gulf [19, 20]. Thus, knowledge of the dinoflagellate endosymbiont genetic “identity” is often critical for reconciling ecological patterns of coral species tolerance to environmental stressors.

Bacterial communities associated with the coral host also promote coral acclimatization/adaptation to changing environmental conditions, including transient stress exposure [16, 23, 24]. Bacteria likely play key functional roles in sustaining nutrient cycling [25] or supporting immunity [23], for example, especially in corals that might otherwise be health compromised. Several studies have reported distinct bacterial taxa associated with corals in extreme habitats such as deepwater [26], volcanic vents [27], and warmer back reef pools [16], suggesting the potential for bacteria to play role in enhancing holobiont environmental plasticity. Transplantation experiments have further demonstrated that bacterial communities shift when corals are introduced to new and non-native habitats, suggesting microbiome alteration as an acclimatization strategy to improve holobiont physiology in response to changing environmental conditions such as salinity, nutrients, and water temperature [16, 17, 28].

Indeed, bacterial communities associated with the coral surface mucus layer (SML) are particularly distinct compared to those associated with the tissue and skeleton [29]. The importance of the SML stems from its protective, nutritional, cleansing roles [30, 31], but notably, it acts as a physical barrier against invasion of potential pathogens [32], therefore forming the first line of defense [30]. Removal of the SML (using antibiotics) caused dramatic necrosis and bleaching with symptoms reflecting the invasion by opportunistic and pathogenic bacteria [33]. Further, the microbiome of coral tissue and skeleton are more influenced by intrinsic factors, unlike the SML bacteria that may be more influenced by environmental variables [34]. As such, changes to the SML bacterial community are more closely tied to environmental variance compared to other coral compartments [17, 23, 32. 35].

The Red Sea represents a unique natural laboratory as it covers 15° latitude and coral conspecifics throughout the Red Sea experience a large environmental gradient, particularly temperature ranging from 23.6 ± 0.6 °C in the north to 29 ± 0.4 °C in the south (mean annual ± SD, see [4]). The susceptibility of these conspecifics to thermal anomalies (i.e., temperatures above the long-term summer mean) is highly variable across latitudinal gradients. For instance, corals in the northern Red Sea experience high thermal anomalies of up to 15 Degree Heating Weeks (DHW) without visible bleaching, in comparison to their central and southern counterparts [4]. This is particularly striking when compared to global patterns of coral temperature vulnerability with mass bleaching most often occurring already after 4 DHW and widespread mortality after 8 DHW [36]. Consequently, the northern Red Sea may represent a refuge where corals exist well below their thermal maxima and are thus likely to be among the last to bleach [4, 37]. Notably, investigation of genetic variability of coral hosts (*Stylophora pistillata* and *Pocillopora verrucosa*) highlighted low genetic difference and weak isolation between populations across the Red Sea, but strong gene flow [38, 39]. Therefore, the association of corals with different microbiome composition may, at least in part, explain holobiont acclimatization to thermal tolerance within the northern Red Sea.

Here, we examined endosymbiont and SML bacterial communities associated with six coral species collected from two environmental settings: (i) across depths to represent different light regimes and (ii) across 4° of latitude (~ 500 km) detailing sites that varied in mean summer temperatures within the northern Red Sea. We characterized the endosymbiont and bacterial composition using high-throughput metabarcoding to determine how, and therefore if, microbial communities are associated with coral acclimatization under different environmental regimes [4]. We show that while the endosymbiotic dinoflagellate communities for a given host are maintained throughout the investigated region, bacterial diversity and composition were site-specific and varied significantly along the latitudinal gradient. These findings suggest that bacterial communities could aid in holobiont acclimatization or adaptation, while the conserved dinoflagellate community may be able to support productivity throughout the northern Red Sea.

## Results

### *Symbiodiniaceae* community structure

**Fig. 1:**
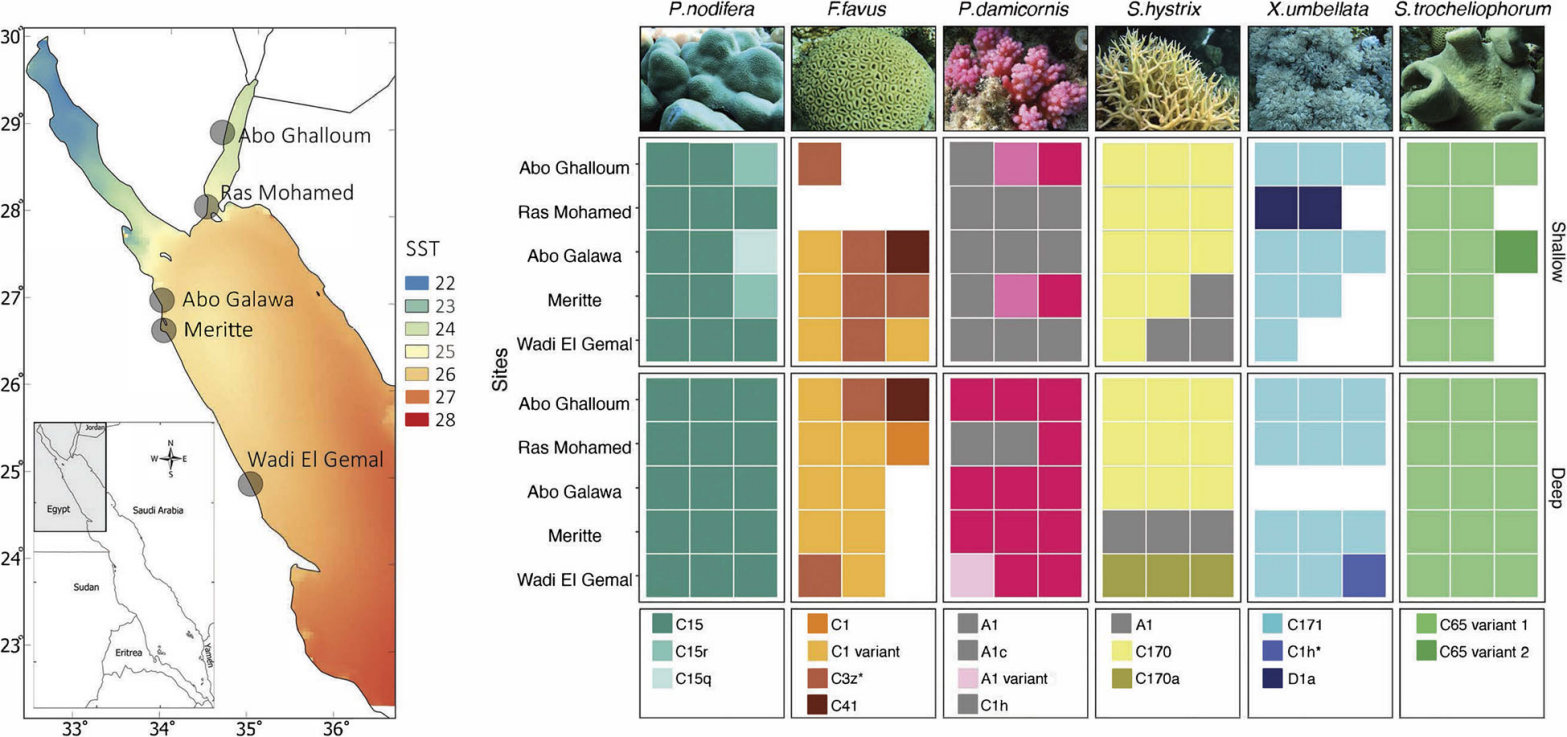
Endosymbiont distribution for six coral species collected from two depths (2–5 m and 15–18 m) along five different sites at the northern Red Sea (total *n* = 163). The map shows the long-term mean of sea surface temperature along the Red Sea and the thermal gradient in the northern Red Sea, including sampling sites. Data obtained from Giovanni Ocean color (https://giovanni.gsfc.nasa.gov/giovanni/, MODIS Aqua 4 km satellite, 4 μm night only) for the period between July 2002 and August 2018. The tile plot represents endosymbiont ITS2 types associated with each coral host, depth, and site separately where site represents a latitudinal gradient (sites on *y*-axis are arranged from the North (top) to South (bottom)). Three distinct patterns are apparent: (i) high degree of host-symbiont specificity, (ii) absence of depth-specific patterns, except for *P. damicornis* and *F. favus,* which changed the ratio of dominant clades with depth, and (iii) symbiont community within each host did not change across the latitudinal gradient, except in *S.hystrix*. White tiles represent missing samples; representative image of coral hosts above tile plot column for each respective species

Samples were collected from six coral species (two species each of branching and massive scleractinian coral and two species of soft coral) to represent taxonomic and functional diversity, at two depths along five sites (*n* = 163) with different thermal regimes in the northern Red Sea (Fig. 1, see [4]). DGGE fingerprinting identified a total of 19 endosymbiotic ITS2 types belonging to three genera: *Symbiodinium*, *Cladocopium*, and *Durusdinium* (from previously described clades A, C, and D, respectively [18]). Endosymbionts from *Cladocopium* were most prevalent (85% of all samples, *n* = 139) and comprised 14 ITS2 types: C1, C1 variant, C15, C15r, C15q, C170, C170a, C171, C1h, C1h*, C3z*, C41, C65 variant1, and C65 variant2 (Fig. 1). The remaining symbionts were from the genus *Symbiodinium* (14%, *n* = 22) and comprised three ITS2 types (A1, A1c, and an A1 variant), and finally *Durusdinium trenchii* D1a (1%, *n* = 2, Fig. 1). Of the 19 symbionts, at least five novel types (i.e., not described previously) were recorded (19%, *n* = 31), namely C15r, C15q in *Porites nodifera*, C3z* in *Favia favus*, and C1h* and C171in *Xenia umbellata* (Additional file 1). Further, four endosymbiont types remained unidentified due to sequencing difficulties (A1 variant, C1 variant, C65 variant1, and C65 variant2).

Overall, the endosymbiont community structure did not vary significantly between the depths (PERMANOVA, *F* = 1.9, *R*^*2*^ = 0.011, *p* = 0.059). Nevertheless, few endosymbiont types were observed only in either samples from the shallow (C15r, C15q, C65 variant2, A1c, and D1a) or deep (C170a, C1, C1h*, and A1variant) (Additional file 2: Figure S1). Despite the latitudinal differences and increase of ambient water temperature toward the south by almost 3 °C, the endosymbiont community structure within each coral host generally did not change between sites (PERMANOVA, *F* = 0.6, *R*^*2*^ = 0.016, *p* = 0.92). Endosymbiont type shifted only in *Seriatopora hystrix* from the dominance of C170 (70%) in the northern sites to A1 (20%) and C170a (10%) types in the southernmost (warmer) sites (i.e., Meritte and Wadi El Gemal—Fig. 1).

The endosymbiont types were strongly linked to coral species identity, indicating a high level of host-specificity (PERMANOVA, *F* = 48.4, *R*^*2*^ = 0.60, *p* < 0.001). Each coral species associated predominantly with either single or multiple distinct endosymbiont type(s) that were rarely shared with other coral species (Fig. 1). The only endosymbiont present across multiple host species was A1, which was recorded in *Pocillopora damicornis* and *S. hystrix* (Fig. 1). Notably, there was no more than one endosymbiont type detected in any of the sampled colonies along the gradient.

*Porites nodifera* associated predominantly with C15, but few colonies contained the novel types C15r (*n* = 1) and C15q (*n* = 2). *F. favus* harbored four types, predominantly an unidentified C1 variant (*n* = 12), the novel C3z* (*n* = 7), C41 (*n* = 2), and C1 (*n* = 1). *P. damicornis* harbored four types, A1 (*n* = 11) and A1c (*n* = 2) in the shallows and mainly C1h (*n* = 12) in the deep—this C1h type is found commonly in pocilloporids across the Indian Ocean [36, 37]—and a single colony with an unidentified variant of *Symbiodinium* A1 (Fig. 1). *S. hystrix* associated with symbionts A1 (*n* = 6), C170 (*n* = 21) and C170a (*n* = 3). The soft coral *X. umbellata* harbored the novel C171 (*n* = 20), two colonies with D1a and a single colony had the novel C1h* type. Finally, *Sarcophyton trocheliophorum* contained two unidentified types closely related to C65 (C65-variant1, *n* = 24 and C65-variant2, *n* = 1), which is a common symbiont found in soft corals on the Great Barrier Reef and Indian Ocean [38, 39]. Thus, coral host identity was the main factor determining endosymbiont variability throughout the latitudinal gradient in the northern Red Sea.

### Bacterial community structure

**Fig. 2:**
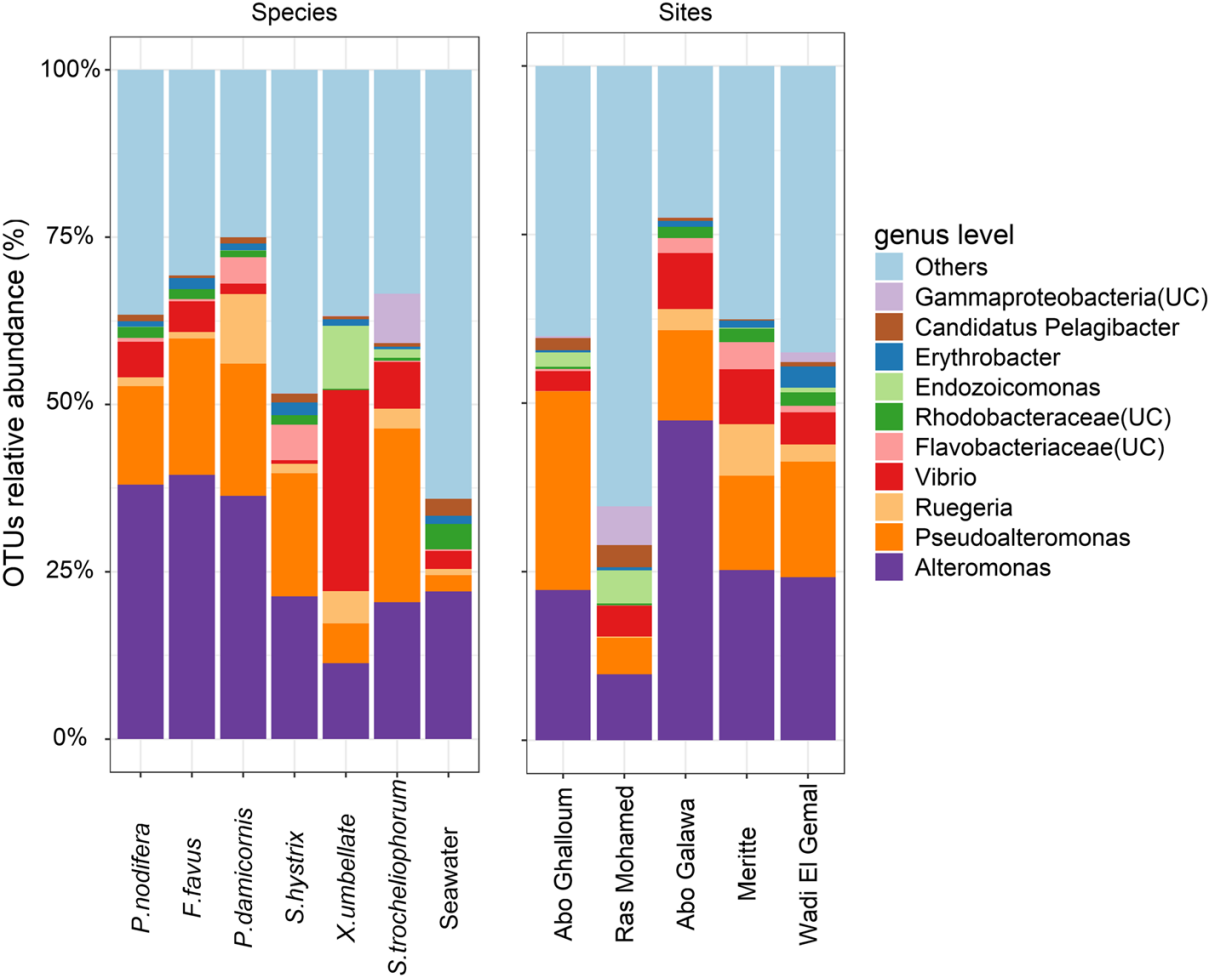
Taxonomic profile (genus level) of the abundant bacterial community associated with the surface mucus layer of six coral species and surrounding seawater samples (left) collected from five surveyed sites (right) in the northern Red Sea. *Alteromonas* and *Pseudoalteromonas* were the most dominant OTUs and composed combined 43.6% of the total community in both sites and coral species, bacterial community was significantly different between sites and coral hosts. Water samples had markedly distinct bacterial assemblage: over 60% of the bacteria had less than 1% of relative abundance. Unclassified taxa to genus level were denoted by (UC)

Bacterial 16S rRNA gene amplicon sequencing from the SML of six coral species and surrounding seawater samples at two depths (*n* = 164) yielded 21.3 million sequences, ranging from 38,048 to 1.3 million sequences per sample (median = 117,188 reads; see Additional file 3). Sequence length ranged from 350 bp to 548 bp (median = 427 bp). A total of 6970 OTUs were recorded across all samples, ranging from 159 to 2556 OTUs per sample (median = 656 OTUs—see Additional file 2: Figure S2). OTUs belonged to 40 bacterial phyla, whereby *Proteobacteria* was the predominant phylum representing 53% of total abundance across all samples (i.e., corals and seawater), followed by *Bacteroidetes* (16%) and unclassified bacteria (10%). Out of 6970 OTUs, only 14 most dominant OTUs comprised 60.9% of the total bacterial community abundance. The remaining OTUs (*n* = 6956) were rare (i.e., each contributed < 1% of total abundance), but shaped the remaining microbial community structure (39.1%) without defined dominant taxa (Fig. 2). The most abundant bacterial phylotypes were a single *Alteromonas* sp. (27.4%) and three *Pseudoalteromonas* OTUs (16.2%), which together comprised 43.6% of the total bacterial abundance of coral SML and seawater (Fig. 2). Three different *Vibrio* OTUs cumulatively comprised 6.6% of all sequences, while *Endozoicomonas* and the photosynthetic *Erythrobacter* were in low abundance (1.2% each). Soft corals appeared to have similar bacterial composition compared to reef-building corals, with *Alteromonas* and *Pseudoalteromonas* comprising the main OTUs; however, *X. umbellata* had relatively high proportions of *Vibrio* and *Endozoicomonas* sp. OTUs (Fig. 2). Similarly, water samples were also dominated by *Alteromonas* sp. (22.1%), but were markedly comprised of different bacterial phylotypes, such as *Roseovarius* sp. (4.6%), *Rhodobacteraceae* (3.8%), and *Pelagibacter* sp. (2.6%) (Table 1).

**Table 1:** Summary of abundant (> 1% of total abundance) and core (present in at least 95% of the samples regardless of abundance) microbial OTUs in the surface mucus layer of six coral species (including water) collected from two depths across the latitudinal gradient in the northern Red Sea

OTUs	Phylum	Class	Genus	Total no. of sequences	Relative abundance %	Core	Abundant
OTU1	*Proteobacteria*	*γ-proteobacteria*	*Alteromonas*	5,851,880	27.4	+	+
OTU2	*Proteobacteria*	*γ-proteobacteria*	*Pseudoalteromonas*	2,208,755	10.4	+	+
OTU94	*Proteobacteria*	*γ-proteobacteria*	*Pseudoalteromonas*	997,177	4.7	+	+
OTU4	*Proteobacteria*	*α-proteobacteria*	*Ruegeria*	722,904	3.4	–	+
OTU15	*Proteobacteria*	*γ-proteobacteria*	*Vibrio*	470,821	2.2	+	+
OTU29	*Proteobacteria*	*γ-proteobacteria*	*Vibrio*	467,620	2.2	–	+
OTU6	*Bacteroidetes*	*Flavobacteriia*	*Flavobacteriaceae*	400,646	1.9	–	+
OTU14	*Proteobacteria*	*γ-proteobacteria*	*Vibrio*	378,498	1.8	+	+
OTU7	*Proteobacteria*	*α-proteobacteria*	*Rhodobacteraceae*	298,855	1.4	–	+
OTU8586	*Proteobacteria*	*γ-proteobacteria*	*Endozoicomonas*	252,966	1.2	–	+
OTU10	*Proteobacteria*	*α-proteobacteria*	*Erythrobacter*	252,405	1.2	+	+
OTU32	*Proteobacteria*	*γ-proteobacteria*	*Pseudoalteromonas*	236,331	1.1	+	+
OTU11	*Proteobacteria*	*γ-proteobacteria*	Unclassified *γ-proteobacteria*	226,023	1.1	–	+
OTU80	*Proteobacteria*	*α-proteobacteria*	*Candidatus Pelagibacter*	221,409	1.0	–	+
OTU12	*Cyanobacteria*	*Cyanobacteria*	*Gplla*	190,784	0.9	+	–

**Fig. 3:**
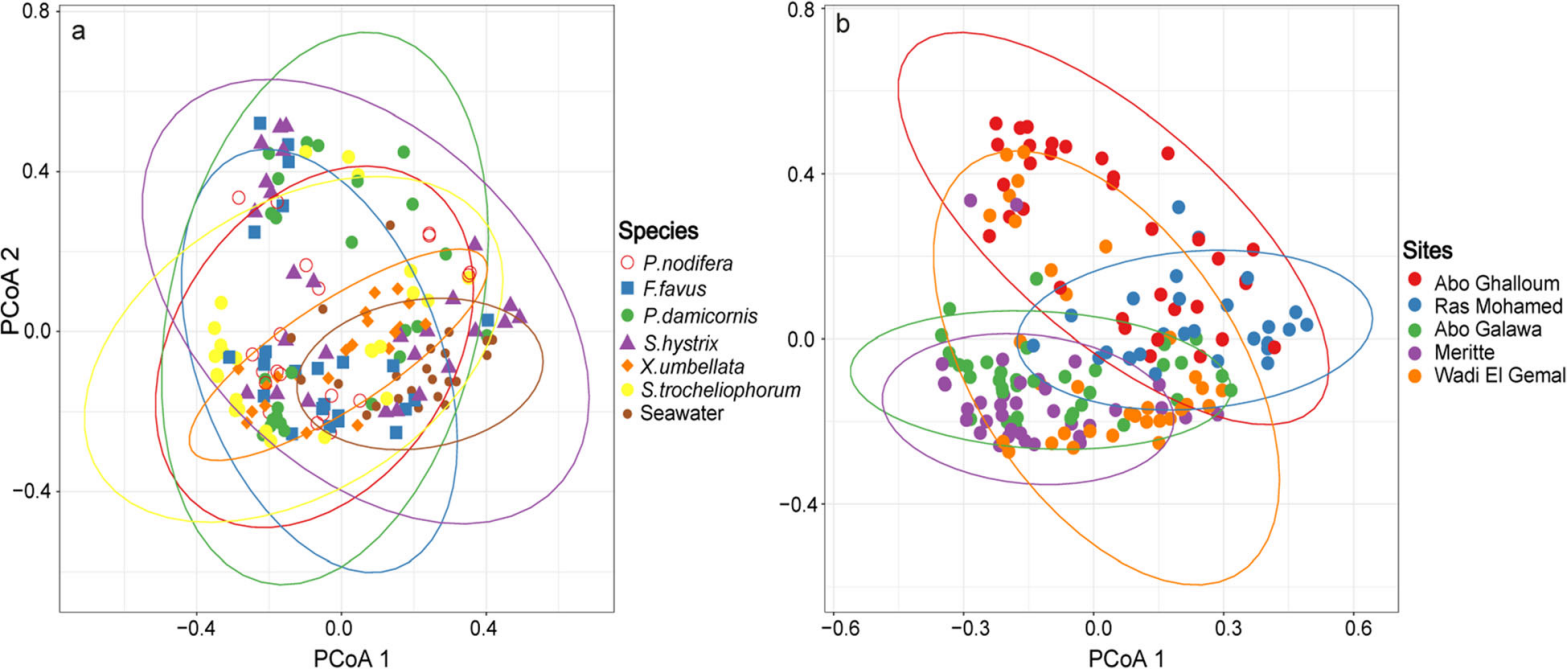
Principal coordinate analysis (PCoA) based on Bray-Curtis dissimilarity matrix of bacterial communities associated with six coral species and five sites along the latitudinal gradient in the northern Red Sea. PCoA shows clustering pattern between coral species versus seawater (**a**) and between different sites (**b**). Two most abundant OTUs (*Alteromonas* sp. and *Pseudoalteromonas* sp.) mask geographic patterns and were therefore excluded for this visualization. Compositional differences in bacterial communities were best explained by site

Seawater samples had a distinct bacterial diversity that was significantly richer (i.e., Chao1–*F*_*1,162*_ = 41.4, *p* < 0.001) and more diverse (i.e., inverse Simpson–*F*_*1,162*_ = 10.7, *p* < 0.01 and Shannon index–*F*_*1,162*_ = 18.7, *p* < 0.001) compared to that of coral SML. Seawater bacterial diversity did not significantly vary with either depth or site (all subsequent ANOVA *p* > 0.05—see Additional file 2: Table S1, Additional file 2: Figure S3). Similarly, the coral SML bacterial richness and diversity did not vary with depth, but in contrast to seawater, differed significantly between sites and coral species (Additional file 2: Table S1). Analysis of bacterial community composition further confirmed this pattern that seawater bacterial communities were significantly different from the coral SML (pairwise PERMANOVA, *F* = 7.2, *R*^*2*^ = 0.04, *p* < 0.001—see Fig. 3). Therefore, seawater samples were removed from subsequent analysis. Similar to bacterial diversity, coral SML-associated bacterial community composition did not vary between depths (PERMANOVA, *F* = 1.4, *R*^*2*^ = 0.01, *p* = 0.14), but by coral host species (PERMANOVA, *F* = 5.3, *R*^*2*^ = 0.168, *p* < 0.01) and site (PERMANOVA, *F* = 8.4, *R*^*2*^ = 0.174, *p* < 0.01). PERMANOVA was also performed on each coral species across all sites separately as well as all coral species within each site, confirming that sites and coral host species contribute to the variation in the bacterial community, but not depth (see Additional file 2: Table S2). Principle coordinate analysis (PCoA) confirmed this pattern (after removal of the two most abundant OTUs, only for this visualization but not excluded from statistical analysis, as they obscured the geographic patterns—see also Additional file 2: Figure S4 for PCoA without removal of those OTUs) and bacterial communities were clustered geographically based on site, regardless of depth and coral species (Fig. 3). However, within each site, bacterial communities were distinct between coral species (Additional file 2: Figure S5 and Additional file 2: Table S2). Thus, bacterial community structure varied with host taxa, similar to dinoflagellate endosymbionts association, but it also differed across the latitudinal gradient.

### Bacterial indicator species

We further performed an indicator species analysis to identify bacterial taxa (OTUs) that are representative of specific sites and coral hosts (cutoff level *p* < 0.05). Due to the similarity between bacterial assemblages at the two depths (PERMANOVA, *F* = 1.4, *R*^*2*^ = 0.01, *p* = 0.14), data were pooled across depths. We found a number of OTUs, ranging from 36 OTUs at Abo Galawa to 1111 OTUs at Ras Mohamed (total 2247 out of 6970 OTUs—32%) that were significantly associated with the site (Additional file 2: Figure S6). The abundance of these OTUs relative to the total microbial community varied from 10.7% at Wadi El Gemal to 58.8% at Ras Mohamed (Additional file 2: Figure S6). The main indicator taxa differed between sites, highlighting the heterogeneity of associated taxa, however, *Pseudoalteromonas* and *Alteromonas* were reported at all sites as indicator OTUs as well (Additional file 2: Figure S7). For example, *Pseudoalteromonas* spp. (69.3%) and *Endozoicomonas* sp. (10%) were the prevalent indicator bacterial OTUs at the northernmost site Abo Ghalloum, but *Psychrosphaera* sp. (23.6%) and *Roseovarius* sp. (15.8%) dominated Meritte, and an unclassified *Gammaproteobacteria* (9.6%), *Endozoicomonas* sp. (8.3%), and *Gplla* sp. (i.e., *Cyanobacteria*—6.3%) dominated Ras Mohamed (Additional file 2: Figure S7). Interestingly, Wadi El Gemal (i.e., the warmest site at the south) was dominated by the photosynthetic *Erythrobacter* sp*.* (29%). Notably, its abundance increase southward aligned with the latitudinal gradient (from 0.3 to 3.1%—Additional file 2: Figure S8).

A total of 977 OTUs (14% of total OTUs) were significantly associated with the SML of different coral hosts, ranging from 26 OTUs in *P. damicornis* to 456 OTUs in *P. nodifera* (Additional file 2: Figure S6). Abundances varied notably between reef-building corals (i.e., *P. nodifera*, *F. favus*, *P. damicornis*, and *S. hystrix*; 5.8% to 18.8%), and soft corals (i.e., *X. umbellata* and *S. trocheliophorum*; 36.6% and 49%, respectively) relative to the total OTUs (Additional file 2: Figure S6). The main bacterial indicator taxa differed between soft coral hosts, revealing species-specific bacteria, with *Pseudoalteromonas*, *Alteromonas*, and *Endozoicomonas* represented in the SML of all investigated host species (Additional file 2: Figure S7).

Linear discriminant analysis (LDA) effect size (LEfSe) analysis supported indicator species analysis and showed that Ras Mohamed was highly enriched by many unclassified bacterial OTUs that drive variation between sites. Overall, 406 OTUs (69 family and 126 genus) were differentially abundant between sites. *Ruegeria*, *Pseudomonas*, unclassified *Flavobacteriacae*, and *Oleibacter* (LDA > 5, *p* < 0.001) were the most significant OTUs that were differentially expressed between sites (Additional file 2: Figure S9a). On the other hand, 380 OTUs (53 family and 97 genus) drove most of the variation between the SML of coral species as well as seawater samples, particularly three *Endozoicomonas* and unclassified *Alteromonadaceae* OTUs that had the highest LDA score (LDA > 5, *p* < 0.001). Interestingly, seawater was enrichened by bacteria that drove most variation between coral species compared to seawater, highlighting the distinct bacterial community of seawater (Additional file 2: Figure S9b).

### Core microbiome of coral SML

**Fig. 4:**
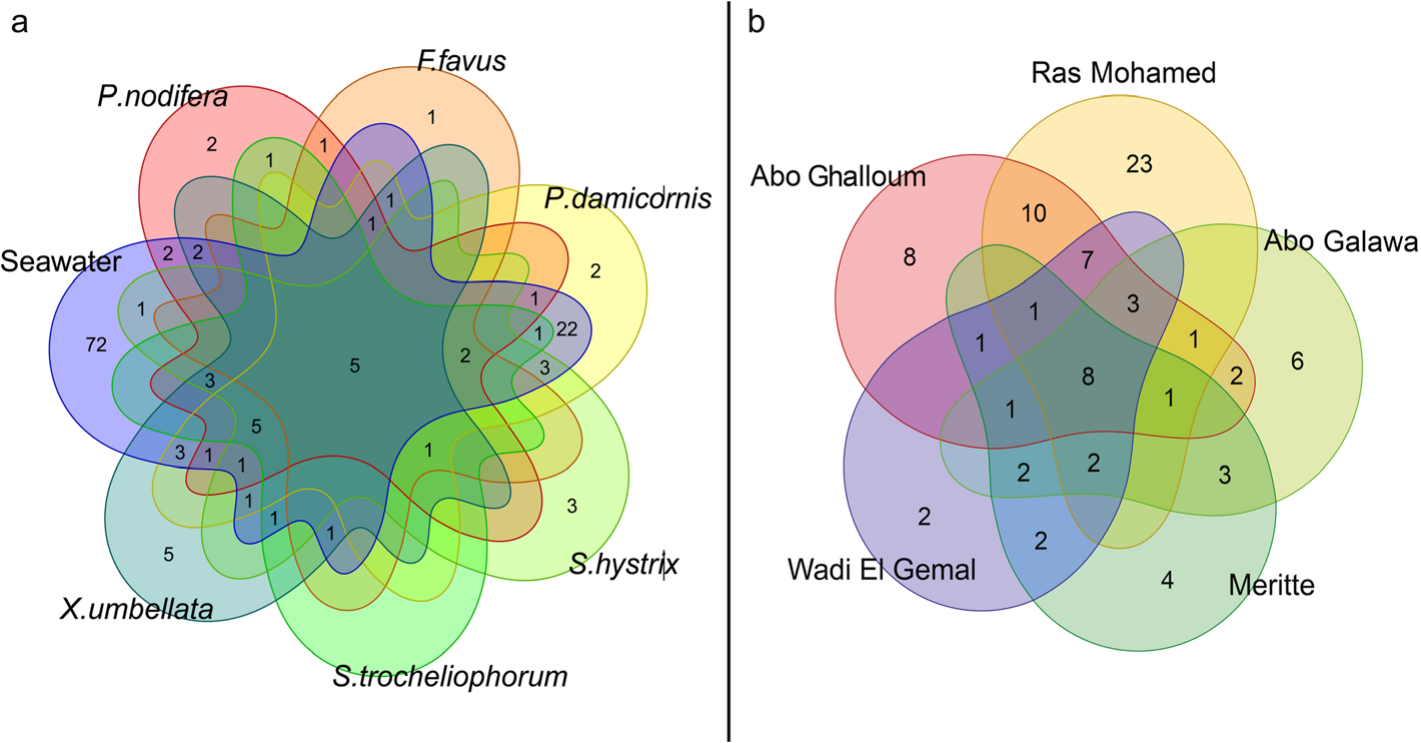
Venn diagram illustrating the number of bacterial OTUs that are present in at least 95% of the samples at each site and coral species. The graph shows the number of core OTUs shared among coral species (**a**). Only five OTUs were common between six corals species and seawater, but seawater samples had 72 exclusive OTUs that were not found in the SML. Similarly, eight OTUs (49.7% of total bacterial abundance) were common between sites (**b**), five of them were shared between all species in addition to a *Vibrio* sp., a *Gplla* sp., and the photosynthetic *Erythrobacter* sp. Importantly, each site and coral species had a small number of exclusive OTUs (outer region in diagram)

The number of core OTUs (i.e., present in 95% of the samples regardless of their abundance) varied between sites and coral species. The total number of core bacteria associated with seawater was 129 OTUs, while it ranged from 13 in *F. favus* to 50 OTUs in *P. damicornis*. Interestingly, only 5 OTUs were common among corals and seawater (*Alteromonas*, 3 OTUs of *Pseudoalteromonas,* and *Vibrio*), but 72 OTUs were exclusively found in the seawater samples highlighting the distinct bacterial community of seawater. Similarly, core bacteria ranged from 56 OTUs at Ras Mohamed to 25 OTUs at Meritte, contributing from 47.3% at Ras Mohamed to 84% at Abo Ghalloum of bacterial abundance (Additional file 2: Table S3). Notably, eight OTUs were shared among all sites (Fig. 4), five of them were the same OTUs shared among all coral species in addition to another 3 OTUs: *Vibrio* sp., *Gplla* sp. (i.e., *cyanobacteria*), and the photosynthetic *Erythrobacter* sp. There were exclusive OTUs in each site that were consistently observed within SML samples across all coral species and ranged from 23 at Ras Mohamed to 2 OTUs at Wadi El Gemal (Fig. 4 and Additional file 4). Interestingly, two exclusive OTUs at Wadi El Gemal (the warmest site) belong to the chemo/phototroph family *Rhodobacteraceae* (Additional file 4), but occurred in low abundance and comprised only 0.3% of total bacterial abundance at this site.

## Discussion

Flexibility of coral microbiomes may help enable coral species to tolerate, and adapt to, environmental conditions known to induce stress and mortality [16, 17, 24, 28, 35. 44]. Here, we assessed the composition of coral dinoflagellate endosymbionts and the SML bacterial community for six abundant coral species inhabiting the northern Red Sea, an area recently proposed as a refuge for corals against thermal stress events [4, 45]. Our data provides new insight into whether and how microbiome composition aligns with the tolerance of corals within a region that is characterized by a natural latitudinal gradient of temperature from north to south and also subjected to extreme thermal anomalies [4].

### Coral host and dinoflagellates endosymbiont associations

Host species identity contributes to endosymbiotic dinoflagellate distribution and diversity [46, 47]. Similarly, we observed a high level of host specificity for each of the coral species and their endosymbiotic dinoflagellates despite the latitudinal gradient, a trend that is consistent with previous studies in different bioregions [40, 42, 48, 49]. Such specificity may be attributed to one or more factors including (i) compatibility of cellular signals between algal symbiont and coral host during symbiosis establishment [50], (ii) metabolic characteristics of the host [22, 51], (iii) availability of host pigments to facilitate photosynthesis [52], (iv) host-driven factors that influence micro-habitat conditions for the symbiont (e.g., colony morphology and tissue thickness which influence light absorption [53], and (v) host acquisition symbiont strategy (vertical versus horizontal transmission—notably, all coral species investigated here were brooders that use vertical transmission) [54]. Environmental conditions are known to shape distribution patterns of the family *Symbiodiniaceae* at larger spatial scales [46, 55, 56]. For example, the main reef builder in the Caribbean (*Orbicella annularis*) exhibits partitioning of the endosymbiont community between the north and south (i.e., 1800 km from the Bahamas to Colombia), which is driven by thermal history rather than host genotype [57]. Thus, flexibility of host-endosymbiont associations (via switching or shuffling) is one strategy for corals to survive across biogeographical regions and under various local environmental stressors [58, 59]. The strong host-endosymbiont associations we observed here likely indicate strong local selection pressure to specific environments of the northern Red Sea [4].

Corals were dominated by *Cladocopium* spp. (formerly clade C, 86%). Presumably, *Cladocopium* spp. dominance in the Red Sea reflects the evolutionary origin (and connectivity) of coral taxa from the Indian Ocean where *Cladocopium* spp. also dominate (see [21, 40, 41, 60–62]). This is consistent with Ziegler et al. [55] who noted *Cladocopium* spp. dominance within the Red Sea (see also Baker et al., [63]) and suggested a strong selection for this genus throughout the entire Arabian region. We found five novel *Cladocopium* types exclusively within the northern Red Sea highlighting endemism within the region. As such, this “thermal refugia area” may select certain symbiont types that broaden the environmental niche for corals to survive under different (and extreme) conditions [4, 11, 12]. Although the phylogenetic analyses of endosymbionts relative to those in the Indian Ocean are yet to be explored, this may highlight the geographically (semi) isolated nature of the Red Sea that would promote local ecological (and/or genetic) speciation of endosymbionts.

The presence of the genus *Symbiodinium* with the genus *Cladocopium* within two branching coral hosts (*P. damicornis* and *S. hystrix*) corroborates that many types of *Symbiodinium* spp. are generalist symbionts within the Red Sea and occupy multiple hosts [64]. This pattern is likely unique for the Red Sea as *Symbiodinium* is rarely recorded in corals elsewhere and usually found in clams and fire corals in the Indo-Pacific Ocean [40].

### Spatial differences of dinoflagellate endosymbiont community

Interestingly, the genetic identity of the algal endosymbionts within a host species did not change from north to south despite the latitudinal gradient. There are two potential explanations for this observation. Firstly, phenotypic plasticity of endosymbionts may enable them to populate different environmental/thermal regimes (e.g., [65]) and therefore influence coral thermal tolerance along the northern Red Sea, possibly via long-term acquisition of endosymbionts locally adapted to high temperature [66]. Howells et al. [67] noted higher photochemical performance and survivorship of *Cladocopium* spp. C1 isolated from warmer areas than those sampled from cooler regimes. Levin et al. [68], subsequently confirmed that this “warmer-regime” *Cladocopium* C1 isolate is characterized by enhanced gene expression for heat tolerance. Thus, the history of environmental regimes are likely critical in expanding niche breadth through functional diversity for certain symbiont taxa [22].

Secondly, the resolution of ITS2 as a genetic marker may underestimate the taxonomic diversity of the family *Symbiodiniaceae*, and as such higher genetic marker resolution is needed to resolve taxonomic diversity [19, 69, 70]. For example, using the *bspA* gene resolved identical C3 ITS2 fingerprints and demonstrated a distinct monophyletic lineage with a large genetic distance of new species (*Cladocopiumthermophilum*) compared to other C3 types from the Arabian Gulf ([19], see also [71]). Notably, this novel species *C. thermophilum* likely consists of different (sub)species [72] that could be resolved using the ITS2 marker alone and the novel analytical framework SymPoral [73]. In our study, we report five novel symbiont types in addition to four unidentified endosymbiont types. This highlights the need to further explore the taxonomy of *Symbiodiniaceae* within the region using different genetic markers that may provide higher taxonomy resolution. Notably, the recent SymPortal framework that resolve symbiont types from ITS2 next-generation sequencing data overcomes many of the recent limitations.

### Bacterial community plasticity across sites

In contrast to the endosymbiont communities*,* the bacterial communities varied primarily between sites highlighting strong geographical patterns, likely driven by environmental differences, over host-bacterial specificity (Fig. 1). Coral bacterial communities are altered by changing environmental conditions such as pollution [17, 74], nutrient availability [17, 75], environmental anomalies such as temperature [16, 21, 76], salinity [28], pH [77, 78], and eutrophication [79]. Such compositional flexibility indicates a potential capacity for local acclimatization (or adaptation), and thus may be critical for promoting holobiont fitness a. Indeed, our indicator species analysis supports the notion of local acclimatization where a high number of OTUs were significantly associated with sites comprising high relative abundance (Additional file 2: Figure S6). This suggests selection of beneficial bacterial taxa that are presumably important to sustain coral survival under different environmental/thermal regimes. Notably, the stark differences that we observed in bacterial communities of the SML along latitudinal gradients are unlikely to be simply explained by differences in the prevailing environment since the bacterial community differences were also host-specific.

### Coral-specific bacteria

Composition of bacterial communities varied between coral species, where 5.8% to 18.8% of the bacterial community associated significantly to specific coral hosts regardless of latitudinal environmental/thermal regimes. Such host-specificity of bacterial communities is consistent with many other studies profiling coral microbiomes [80–83]. The variation in bacterial community composition between coral taxa could be explained by different factors including (i) species-specific composition of exudates by different coral hosts to the SML (e.g., [84]), (ii) species-specific biochemical composition of coral SML itself [85–88]; (iii) vertical transmission of bacterial community from parents to offspring [89–92]. However, which of these factors likely drive differences among bacterial communities of northern Red Sea corals is difficult to discern from the available data.

### Putative roles of the dominant SML-associated bacteria

SML bacterial community functional characterization is required to validate whether and how bacterial taxa contribute to niche broadening throughout the northern Red Sea and is beyond the scope of the curernt study [93]. Even so, using the METAGENassist web portal [94] (Additional file 2: Figure S10), functional profiling showed that about half of the samples exhibited potential enrichment of high sulfate and nitrite reducers and dehalogenating bacteria, while the remaining samples had mixed metabolic sources. High enrichment of sulfate reducers may simply reflect the dominance of *Alteromonas* and *Pseudoalteromonas*, many of which are known to play a major role in dimethyl-sulfoniopropionate (DMSP) metabolism [95]. High abundance of these genera may indicate elevated levels of DMSP production in Red Sea corals and surrounding waters [96]. DMSP is produced by the endosymbiotic dinoflagellates as well as the coral [97] and metabolized by associated bacteria to form dimethylsulfide (DMS), dimethyl sulfoxide (DMSO), and acrylate. These molecules have potential roles in osmoregulation [98] and antioxidant capacity [99, 100]. High production of DMSP may therefore convey the capacity to survive under high salinity and thermal anomalies that characterize the northern Red Sea [4, 11, 12]. Similarly, the SML of *Porites lobata* from the central Red Sea was dominated by *Pseudoalteromonas* [101] and displayed increased levels of sulfur cycling compared to the Arabian Gulf. Therefore, it would be informative to link levels of DMSP (and associated by-products) with tolerance patterns of corals in this region.

Interestingly, the presence of *Alteromonas* and *Pseudoalteromonas* within the region may be linked to coral disease resistance. *Pseudoalteromonas* spp. associated with the SML of several coral species exhibit extracellular antibacterial metabolites which may aid in the defense against invasive pathogens [102, 103]. Wright et al. [104] similarly reported an increased abundance of *Alteromonas* and *Pseudoalteromonas* phylotypes in *Acropora millepora* resisting infection by *Vibrio* sp. Further, some strains of *Alteromonas* spp. are known dinitrogen fixers, which may subsequently translocate fixed nitrogen to the algal endosymbionts associated with *P. damicornis* larvae [92]. Together, these observations highlight that while the detailed role of abundant bacterial phylotypes is yet to be investigated for Red Sea corals, they may provide several functions important to holobiont fitness.

Furthermore, *Erythrobacter* sp. constituted the dominant indicator taxon at the warmest site (i.e., Wadi El Gemal) where it increased in abundance southwards (Additional file 2: Figure S8). This bacterial group contains bacterial chlorophyll-*a* (B-Chl*a*) and large amounts of carotenoids [105–108]. Carotenoids are well known for their antioxidant activities [109], but the relationship between bacterial assemblage associated with coral SML and antioxidant activities is not well resolved. Diaz et al. [110] experimentally measured extracellular superoxide concentrations associated with corals and revealed that the microbial community could produce and importantly regulate ROS in their immediate surroundings, and hence influence coral physiology and health. Hence, *Erythrobacter* may play a functional role critical toward improving holobiont resistance to heat stress: however, such functional implications have yet to be fully assessed.

## Conclusion

We provide the first details of the microbiome communities associated with coral conspecifics across 500 km in the northern Red Sea that showed high thermal tolerance as evidenced by low bleaching susceptibility over the past three decades. Our data highlight that the endosymbiotic communities were highly host-specific with little variation throughout this region. At the same time, we identified five novel types highlighting endemism and the selection of certain genotypes within the region. In contrast, the SML bacterial communities varied significantly between sites and coral hosts, therefore emphasizing how the holobiont composition changes across the latitudinal gradient. Among the associated bacterial OTUs, we identified taxa across the northern Red Sea that may play a role in elevated thermal tolerance and may fill a regional environmental niche that broadens the capacity of corals to survive under extreme conditions. We therefore conclude that the distinct microbiome associated with corals from the northern Red Sea may contribute to the thermal tolerance of corals, previously denoted as a coral reef refuge. Notably, the highly responsive nature of bacterial communities present in the SML provides further justification to investigate their functional role, which may contribute to the success of corals experiencing an increased frequency of thermal stress in the near future.

## Materials and methods

### Survey sites and sample collection

#### Sampling sites

Sample collection was conducted at five sites along the northern Gulf of Aqaba and southern Egyptian coast in February 2013, representing a latitudinal gradient mainly varied in temperature. The selected sites were Abo Ghalloum (28.6147°N, 34.5604°E; Gulf of Aqaba), Ras Mohamed (27.7305°N, 34.2691°E; Sinai Peninsula), (3) Abo Galawa (27.3157°N, 33.8097°E), (4) Meritte (27.2485°N, 33.849°E) at Hurghada, and (5) Wadi El Gemal (24.6988°N, 35.1327°E) at the southern Egyptian coast (Fig. 1). All sampling sites were unurbanized and not directly impacted by anthropogenic activities (except Merritte) and characterized by fringing reefs adjacent to the shoreline, except for Abu Galawa which was a patch reef located about 1.5 km off the coast. Sites were located within the thermal gradient where long-term mean (±SD) of summer SST ranged from 26.6 ± 1 °C for the Gulf of Aqaba to 29.3 ± 1.2 °C for the Wadi El Gemal [4]. To ensure that the influence of seawater parameter on coral microbiome composition is minimum, remote sensing data (2003–2012) of chlorophyll *a* and water attenuation coefficient were used as proxy of water quality. This data showed that there are no significant differences in environmental variables across sites, but the temperature is systematically changing across the region (Additional file 2: Supplementary material). Further, we collected seawater samples to measure ammonia concentration in each study sites, that did not vary significantly between sites (Additional file 2: Supplementary material). Taken together, this highlights that temperature is likely the main driver of compositional change of microbiome along the latitudinal gradient in the northern Red Sea.

#### Sample collection

Six coral species were sampled at each site across the latitudinal gradient, with the selection of species representing different coral growth forms: massive *(Porites nodifera*, *Favia favus*) and branching (*Pocillopora damicornis*, *Seriatopora hystrix*) hard coral, as well as soft corals *(Xenia umbellata*, *Sarcophyton trocheliophorum*). Specimens were collected from shallow (2–5 m) and deep (15-18 m) reef slopes representing different light regimes as per Kuguru et al., [111]. At each site, three types of samples were collected: coral fragment, coral mucus and seawater. Overall, three replicates x five sites x six species x two depths samples of coral tissue (total *n* = 164) and coral SML (total *n* = 141) were collected for endosymbiont ITS2 and bacterial 16S rRNA gene profiling, respectively. Further, three replicates of water samples ×  2 depth × 5 sites (total *n* = 23) were also collected as reference bacterial samples. Specifically, (i) coral fragments (< 1 cm) were collected from three different visibly healthy colonies (> 5 m apart) for each species and depth (i.e., *n* = 3 per species and depth). Samples were sealed in separate pre-labeled bags filled with in situ seawater [48]. (ii) At each sampled coral colony, associated SML was sampled using sterile 50 ml syringes (*n* = 3 per species and depth). (iii) Seawater samples (500 ml) were collected in sterilized polyethylene bottles in each site at each depth (*n* = 3 per site per depth) as environmental bacterial reference samples [102]. All samples were then kept shaded in a cold box until preservation (within 2 h).

Upon return to the laboratory, all coral fragments were preserved directly in pre-loaded 2 ml vials containing DMSO-20% buffer for DNA preservation for subsequent dinoflagellate endosymbionts identification [112]. Each SML and water sample was filtered through sterilized 0.22 μm Cyclopore filter columns (Whatman, UK), and preserved in 2 ml vials preloaded with DMSO-20% buffer for 16S rRNA gene microbial analysis. Preserved coral fragments and filtered bacterial samples were kept at 4 °C until shipping to the UK for genomic analysis, and then stored at − 20 °C.

### *Symbiodiniaceae* identification

The overall purpose of our analysis was to retrieve the dominant endosymbiont type(s). For this reason, we determined DGGE to be the most cost-effective approach that can detect up to 90–95% of the total community present within a single coral colony [113]. Notably, DGGE is not a method to elucidate fine-scale genetic differentiation, which is rather conducted via next-generation sequencing of the ITS2 marker gene [114] and subsequent analysis in SymPortal [73]. Endosymbiont DNA was extracted from approximatly 100 mg of coral tissue using the modified Promega Wizard DNA prep protocol (Madison, WI, USA) as per LaJeunesse et al. [48]. Amplification of the symbiont Internal Transcribed Spacer (ITS2) was performed against a negative control, through two steps as described by Bongaerts et al. [105]: (i) nested PCR was used (10 μl total reaction) to amplify the region between 18S and 28S rDNA (750 bp) using 1 μl of gDNA mixed with “ZITSUPM13” and “ZITSDNM13” primers for 35 cycles as described in Santos et al. [116] (Additional file 2: Table S1); and subsequently (ii) 1 μl of the nested PCR amplicon served as a template to amplify ITS2 (330–360 bp) mixed with ‘ZITS2for’ and GC clamp ‘ZITS2 clamp’ primers as designed by LaJeunesse and Trench [117], and touchdown PCR protocol for 40 cycles were used as per LaJeunesse et al. [43] (Additional file 2: Table S1). ITS2 amplicons were then separated by denaturation gradient gel electrophoresis (DGGE) (45–80% polyacrylamide gel) and aligned against a reference DNA ladder (containing ITS2 *Breviolum* B1, *Cladocopium* C1, and *Durusdinium* D1 samples) at 60 °C for ~ 15 h as per LaJeunesse [48] using a CBS Scientific System (Del Mar, CA, USA). DGGE gels were stained with SYBR green (Molecular Probes, Eugene, OR, USA) and representative bands (*n* = 3–5 from different samples from each fingerprint found) for each coral species were excised and eluted in 500 μl RNase free water at 4 °C overnight. Subsequently, bands are directly amplified (without gel extraction step) using ZITS2 forward and reverse primers (without the GC clamp) for 30 cycles and sent for sequencing. After that, the ITS2 amplicon was cleaned using USB-EXO SAP-IT PCR cleanup kit (Affymetrix, USA) and sequenced using Applied Biosystems 310 genetic analyzer, USA.

### Bacterial 16S rRNA gene profiling

Due to the rapidly adaptive nature of the surface mucus layer (SML) to local environments and/or stress, bacterial genomic DNA was extracted from coral SML and seawater using the CTAB (Cetyl-trimethyl-ammonium-bromide) method [119]. To amplify the bacterial 16S rRNA gene from SML and water samples, hypervariable regions V3 and V4 of ribosomal DNA were targeted (~550pb) using 341F and 805R universal bacterial primers with an Illumina overhang adaptor (Additional file 2: Table S1) according to the manufacturer’s protocol (Illumina, San Diego, CA, USA). The PCR amplicon was cleaned by an AMPure XP magnetic bead system (Beckman Coulter, Brea, CA, USA), and 5 μl of cleaned PCR amplicon used for indexing PCR using Nextera XT V2 kit (A&B index kit) (Illumina) according to the manufacturer’s protocol. The indexed PCR amplicon was cleaned again by AMPure XP magnetic beads and then quantified using a FLUOstar Omega microplate reader (BMG Labtech, Germany) using Quant-iT PicoGreen dsDNA assay kit (Invitrogen, USA). All samples were then pooled in equimolar ratios. The quality of the final pooled library was checked on a 1% agarose gel as well as on a Bioanalyzer (Agilent 2100, Santa Clara, CA, USA). Version 3 chemistry kit was used in HiSeq and sequencing was conducted at the TGAC genomic analysis center (Norwich, UK).

### Data analysis

#### Symbiodiniaceae analysis

We followed the commonly accepted and widely published protocols for this technique to interpret this type of data (cf. original methodologies in LaJeunesse et al. 2002 [118]). First, symbiont DGGE gels were assessed visually to identify the fingerprint for each coral sample (Additional file 6), and then DNA sequences for representative bands were obtained, trimmed manually, aligned using Geneious (V10), and then blasted against Genbank ‘nr’ database (http://www.ncbi.nlm.nih. gov/BLAST/) for ITS2 type identification. Each identified ITS2 type was tabulated and transformed into presence/absence data matrix for statistical analysis. To test the significance of similarity of symbiont community between sites, coral species, and depth, we performed Permutation Multifactorial Analysis of Variance (PERMANOVA) [120] with 9999 permutations using Jaccard dissimilarity matrix by *“adonis”* function in R [121] using vegan package in R. Notably, the nature of symbiont community dataset was “presence/absence” while bacterial community was “abundance-based” dataset and therefore they were analyzed separately.

#### Bacterial bioinformatic analyses

Raw 16S rRNA gene amplicon sequences were trimmed using Sickle version 1.33 [122] at the default quality threshold (Q20) using the paired-end mode. Sequence trimming was performed at the 3’ end, and to ensure high taxonomic resolution, all sequences shorter than 350 bp or having ambiguous bases (Ns) were discarded. The forward and reverse sequences that passed quality filter were then subjected to error correction using Bayes Hammer implemented in SPAdes v3.7.1 with default settings [123, 124]. Paired-end sequences were aligned and primers removed using the PEAR algorithm implemented in PANDAseq version 1.33 [125, 126]. Chimeric check was performed using RDP 16S rRNA gene database to ensure sequences quality [117], and paired reads were then de-replicated, sorted by abundance, and clustered into operational taxonomic units (OTUs) at 97% similarity threshold using VSEARCH v1.11.1 (Rognes, https://github.com/torognes/vsearch). Low abundance sequences (< 5 occurrences over all samples) and non-bacterial OTUs (i.e., mitochondria, chloroplast, archaea, eukaryote, and unknown sequences) were then removed. Taxonomic divisions were assigned as OTU centroids using the RDP classifier [127] as implemented in QIIME [128], with a minimum confidence level of 0.7, and relative abundances of taxa were computed using QIIME’s “summarize_taxa.py” script.

#### Bacterial community analysis

The OTU abundance matrix of the microbial community (using non-normalized approach [129]—see Additional file 5) was used to calculate microbial diversity indices (i.e., Chao1 richness estimator, inverse Simpson, and Shannon diversity indices) for each coral sample (total *n* = 164). Normality of diversity indices outcome was checked using the Shapiro test [130], and log-transformed to assess the influence of site, coral species, and depth on microbial diversity using multifactorial ANOVA. The bacterial communities associated with soft coral species (*X. umbellata* and *S. trocheliophorum*) appeared similar in diversity and composition to those associated with reef-building corals (see Additional file 2: Figure S3), and therefore soft corals were included in the remaining analysis.

Multivariate analysis was further used to test the statistical difference of microbial community structure. Permutation Multifactorial Analysis of Variance (PERMANOVA) [120] with 9999 permutations using Bray-Curtis dissimilarity matrix by *“adonis”* function in R was performed on (i) all coral samples to assess the influence of site, coral species, and depth and their interactions on microbial community structure, (ii) on each coral species across sites to investigate the effect of site on each coral species separately, and (iii) on each site to include all coral species (i.e., all corals within each site) to assess the influence of coral species on microbial composition at each site separately. Principal coordinate analysis (PCoA) ordination based on Bray-Curtis dissimilarity was used to visualize the dispersion of microbial community among sites, coral species, and depth.

Indicator species analysis was performed to test the association between bacterial community and between both, sites and coral host, using indicspecies package in R [131]. Linear discriminant analysis (LDA) effect size (LEfSe) analysis was also performed to obtain the most differentially abundant bacteria between sites and coral species using the Microbiome Analysis web portal (https://www.microbiomeanalyst.ca/) with default settings [132]. To investigate OTUs that were consistently associated with coral SML and whether/how they changed with the increase of the ambient temperature across sites, data were transformed into a presence/absence data matrix, and the core mucus microbiome calculated as the occurrence of each OTU in 95% of the samples (i.e., 95% occurrence threshold) across sites. All plots and statistical analysis were performed in R version 3.2.3 [133].

## Supplementary information

**Additional file 1.** Novel ITS2 Sequances.

**Additional file 2: ** Supplementary methods, figures and tables.

**Additional file 3.** Bacterial OTU table.

**Additional file 4.** Core microbiome.

**Additional file 5.** Normalization and-beta-diversity.

**Additional file 6.** Endoymbiont DGGE fingerprints.

## Data Availability

The generated datasets for the current study are available as Additional files 1, 2, 3, 4, 5, and 6, while all raw sequences are available as NCBI BioProject PRJNA509355. GenBank accession numbers for novel endosymbionts are MN968212 to MN968217.
