# Correction to: Coral microbiome composition along the northern Red Sea suggests high plasticity of bacterial and specificity of endosymbiotic dinoflagellate communities

**DOI:** 10.1186/s40168-020-00807-y

**Published:** 2020-02-21

**Authors:** Eslam O. Osman, David J. Suggett, Christian R. Voolstra, D. Tye Pettay, Dave R. Clark, Claudia Pogoreutz, Eugenia M. Sampayo, Mark E. Warner, David J. Smith

**Affiliations:** 1 Coral Reef Research Unit, School of Life Sciences, University of Essex, Colchester, CO4 3SQ UK; 2 Marine Biology Department, Faculty of Science, Al-Azhar University, Nasr City, Cairo, 11448 Egypt; 3 Climate Change Cluster, University of Technology Sydney, Sydney, New South Wales 2007 Australia; 4 Red Sea Research Center, Division of Biological and Environmental Science and Engineering (BESE), King Abdullah University of Science and Technology (KAUST), Thuwal, Saudi Arabia; 5 Department of Biology, University of Konstanz, 78457 Konstanz, Germany; 6 School of Marine Science and Policy, College of Earth, Ocean, and Environment, University of Delaware, Lewes, DE 19958 USA; 7 ARC Centre of Excellence for Coral Reef Studies, School of Biological Sciences, The University of Queensland, St. Lucia, QLD 4072 Australia

**Correction to: Microbiome**

**https://doi.org/10.1186/s40168-019-0776-5**

Following publication of the original article [1], the authors reported an error on the legend of of P.damicornis in Fig. 1. It is all grey instead of grey and pink gradients. The correct figure is presented here.

**Fig. 1:**
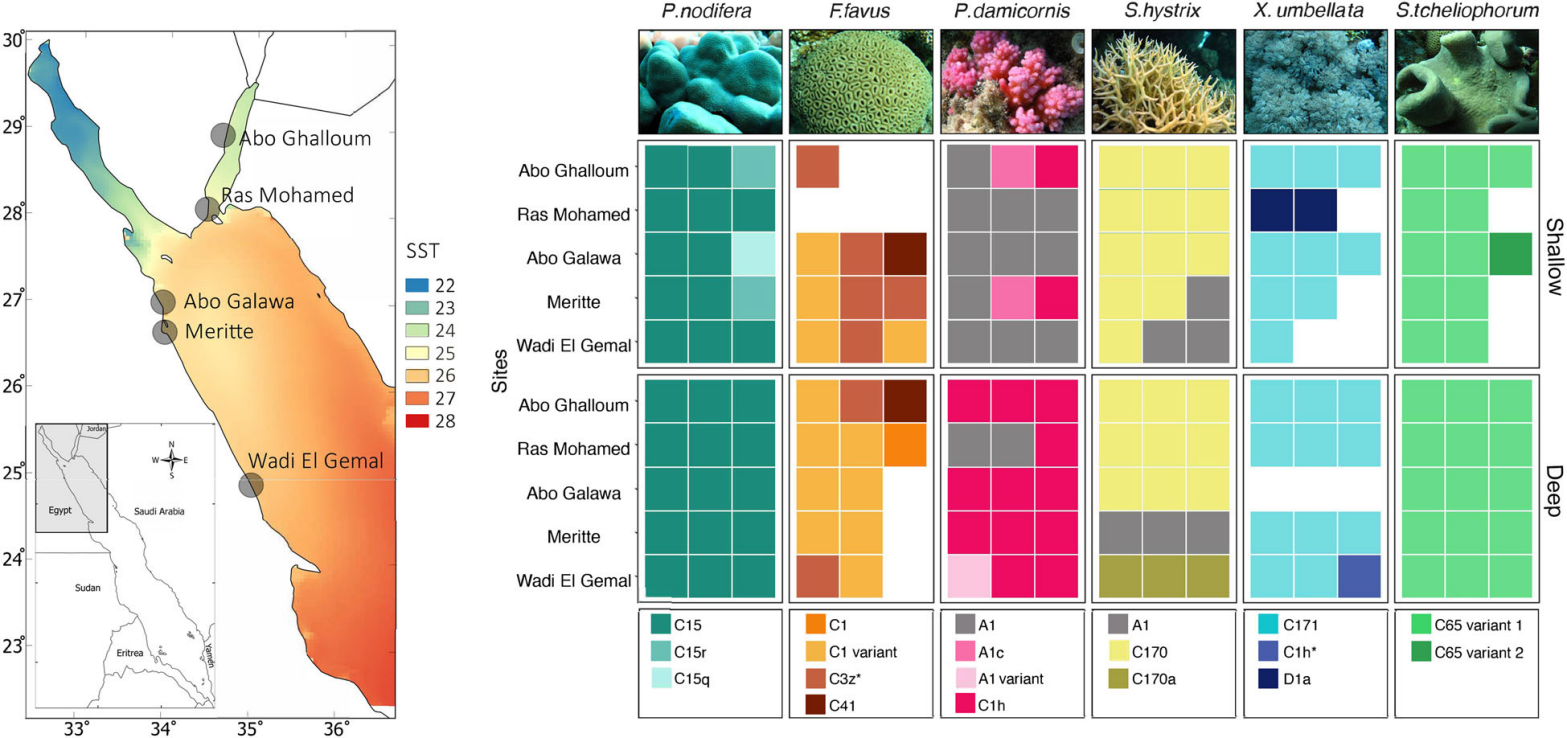
Endosymbiont distribution for six coral species collected from two depths (2–5 m and 15–18 m) along five different sites at the northern Red Sea (total *n* = 163). The map shows the long-term mean of sea surface temperature along the Red Sea and the thermal gradient in the northern Red Sea, including sampling sites. Data obtained from Giovanni Ocean color (https://giovanni.gsfc.nasa.gov/giovanni/, MODIS Aqua 4 km satellite, 4 μm night only) for the period between July 2002 and August 2018. The tile plot represents endosymbiont ITS2 types associated with each coral host, depth, and site separately where site represents a latitudinal gradient (sites on y-axis are arranged from the North (top) to South (bottom)). Three distinct patterns are apparent: (i) high degree of host-symbiont specificity, (ii) absence of depth-specific patterns, except for *P. damicornis* and *F. favus*, which changed the ratio of dominant clades with depth, and (iii) symbiont community within each host did not change across the latitudinal gradient, except in S.hystrix. White tiles represent missing samples; representative image of coral hosts above tile plot column for each respective species
