# Supplementary material for: Coral microbiome composition along the northern Red Sea suggests high plasticity of bacterial and specificity of endosymbiotic dinoflagellate communities
Source: Microbiome. 2020 Feb 6;8:8. doi: 10.1186/s40168-019-0776-5 (PMC6996193; doi:10.1186/s40168-019-0776-5)
Supplement: Supplementary file 2 — Additional file 2: Supplementary methods, figures and tables. [file 40168_2019_776_MOESM2_ESM.pdf]

Additional file

Coral microbiome composition along the northern Red Sea suggests high plasticity of bacterial and specificity of endosymbiotic dinoflagellate communities

Eslam O. Osman[1,2*], David J. Suggett[1,3], Christian R. Voolstra[4,5], D. Tye Pettay[6], Dave R. Clark[1], Claudia Pogoreutz[4,5], Eugenia M. Sampayo[7], Mark E. Warner[6], David J. Smith[1]

[1] Coral Reef Research Unit, School of Biological Sciences, University of Essex, CO4 3SQ, UK

[2] Marine Biology Department, Faculty of Science, Al-Azhar University, Nasr City, Cairo, 11448, Egypt

[3] Climate Change Cluster, University of Technology Sydney, New South Wales, 2007, Australia

[4] Red Sea Research Center, Division of Biological and Environmental Science and Engineering (BESE), King Abdullah University of Science and Technology (KAUST), Thuwal, Saudi Arabia

[5] Department of Biology, University of Konstanz, Konstanz 78457, Germany

[6] School of Marine Science and Policy, College of Earth, Ocean, and Environment, University of Delaware, Lewes, DE 19958, USA

[7] ARC Centre of Excellence for Coral Reef Studies, The University of Queensland, St. Lucia, QLD 4072, Australia

*Corresponding author: Eslam O. Osman

Email: eom.osman@gmail.com

Table S1. Summary statistics of ANOVA performed on the outcomes of richness estimator and diversity indices to test the difference of microbial community among six coral species and water samples along five surveyed sites and two different depths. The water samples were separated from coral mucus to test the variability microbial diversity among corals species themselves without influence of reference water samples. Statistics revealed that microbial diversity changed among depth, but mainly between coral species and sites.

	**Factor/ diversity indices**		**Choa1 estimator**		**Inverse Simpson**		**Shannon**	
		Df	F	P value	F	P value	F	P value
**Corals**	Depth	1	2.2	p>0.54	1.7	p>0.14	3.7	p>0.06
	Sites	4	12.6	p<0.001***	16.7	p<0.001***	28.5	p<0.001***
	Coral Species	5	12.9	p<0.001***	4.1	P<0.001***	8.5	p<0.001***
	Depth*Sites	4	6.2	p<0.001***	2.3	p>0.06	5.1	p<0.001***
	Depth*Species	5	5.4	p<0.001***	1.2	p>0.28	1.9	p>0.08
	Sites*Species	20	2.5	p<0.01**	2.1	p<0.01**	3.4	p<0.001***
	Depth*Sites*Species	16	1.6	p>0.56	1.2	p>0.30	1.6	p>0.06
**Water**	Depth	1	4.2	p>0.06	0.3	p>0.57	0.7	p>0.41
	Sites	4	2.6	p>0.09	1.9	p>0.17	2.1	p>0.13
	Depth*Sites	4	1.9	p>0.15	0.3	p>0.88	0.9	p>0.48

Table S2. Statistical summary of permutation multivariate analysis of variance (PERMANOVA, permutation level 9999) performed on microbial community associated with each site (i.e. all corals species within each site) and coral species (i.e. each corals species across sites) separately based on Bray-Curtis dissimilarity matrix. Multifactorial analysis (PERMAONVA) performed to investigate the influence of site and depth on each coral species, while influence of corals species and depth at each site and their interactions on microbial community composition.

**Variable / Analysis**		**PERMANOVA**					
		DF	Sum of Sqs	Mean Sqs	F Model	R^2	P value
Porites	Depth	1	0.17	0.17	1.26	0.042	0.25
	Site	4	1.532	0.383	2.827	0.373	0.001 ***
	Depth * Site	3	0.915	0.305	2.250	0.222	0.01 **
Favia	Depth	1	0.151	0.151	1.185	0.031	0.28
	Site	4	2.614	0.654	5.137	0.533	0.001 ***
	Depth * Site	3	0.363	0.121	0.951	0.074	0.5
Pocillopora	Depth	1	0.340	0.340	2.534	0.059	0.03 *
	Site	4	2.682	0.670	5.048	0.471	0.001 ***
	Depth * Site	3	0.542	0.180	1.361	0.095	0.16
Seriatopora histrix	Depth	1	0.200	0.200	1.210	0.027	0.26
	Site	4	2.850	0.715	4.338	0.386	0.001 ***
	Depth * Site	3	1.552	0.388	2.355	0.209	0.001 ***
Sarcophyton	Depth	1	0.224	0.224	1.455	0.034	0.16
	Site	4	3.077	0.769	4.990	0.467	0.001 ***
	Depth * Site	3	0.977	0.244	1.584	0.148	0.049 *
Xenia	Depth	1	0.520	0.520	2.874	0.086	0.01 *
	Site	4	2.521	0.630	3.488	0.417	0.001 ***
	Depth * Site	3	0.843	0.281	1.555	0.139	0.07

Microbial commuinity change between corals species among each site

		DF	Sum of	Mean	F	R^2	P value
Abo Ghalloum	Depth	1	0.217	0.217	1.604	0.032	0.13
	Coral species	5	3.081	0.616	4.559	0.452	0.001 ***
	Depth* Species	3	0.956	0.239	1.140	0.140	0.05 *
Ras Mohamed	Depth	1	0.421	0.421	2.502	0.074	0.01 *
	Coral species	5	2.518	0.504	2.993	0.442	0.001 ***
	Depth* Species	3	0.744	0.248	1.131	0.131	0.05
Abo Galawa	Depth	1	0.167	0.167	1.370	0.031	0.22
	Coral species	5	2.194	0.439	3.603	0.412	0.001 ***
	Depth* Species	4	0.529	0.132	1.087	0.099	0.35
Meritte	Depth	1	0.313	0.313	1.736	0.043	0.07
	Coral species	5	2.747	0.550	3.051	0.381	0.001 ***
	Depth* Species	5	0.903	0.181	1.004	0.125	0.47
Wadi El Gemal	Depth	1	1.283	1.283	8.500	0.164	0.001 ***
	Coral species	5	2.656	0.531	3.518	0.339	0.001 ***
	Depth* Species	5	1.487	0.297	1.969	0.189	0.001 ***

Table S3. Summary of total sequences and percentage of core microbiome that present with at least 95% of the samples of each sites.

Site	Total reads	Core reads	Abundance %	No. of OTUs
**Abo Ghalloum**	3899790	2786614	71.5	43
**Ras Mohamed**	2760068	1308085	47.4	56
**Abo Galawa**	4716323	3943787	83.6	29
**Meritte**	6113019	4162589	68.1	25
**Wadi EL Gemal**	3838825	2356267	61.4	29

Table S4. Summary of primers and PCR protocols used in the study to amplify the ITS2 region in Symbiodiniaceae and V3-V4 region within the 16S rRNA gene in bacteria.

	Primer code	Sequence	PCR protocol
**18S and 28S rDNA**	ZITSUPM13	5´CACGACGTTGTAAAACGACCCGGTG AATTATTCGGACTGACGCAGT GCT 3´	94°C for 2 min initial denaturation, 35 cycles at 94°C/30sec, 60°C/30sec, 72°C/45sec, and final extension at 72°C for 7 min
	ZITSDNM13	5´GGATAACAATTTCACACAGGCT GTTTAGTTCCTTTTCCTCCGC 3´	
**ITS2 amplification**	ZITS2for	5´GAATTGCAGA ACTCCGTG 3´	Annealing conditions were 10°C above the final annealing temperature (62°C) and decreased by 0.5°C at first 20 cycles to reach 52°C, and then run 20 cycles at 90°C/30sec, 52°C/40sec, 72°C/30 sec and final extension at 72°C/10 min.
	ZITS2 clamp (underlined)	5´ CGCC CGCC GCGC CCCG CGCC CGTC CCGC CGCC CCCG CCCG GGAT CCAT ATGC TTAA GTTC AGCG GGT 3´	
**16S rRNA gene**	341F forward primer with illumina overhang adaptor (underlined)	5' TCGT CGGC AGCG TCAG ATGT GTAT AAGA GACAG CCT ACGG GNGG CWGC AG 3'	95°C for 3 minutes followed by 33 cycles of: 95°C/30sec, 55-58°C/30 sec (as needed to avoid primer dimer), 72°C/30sec, and finally 72°C/5 mins as final extinction time
	805 reverse primer with illumina overhang adaptor(underline)	5' GTCT CGTG GGCT CGGA GATG TGTA TAAG AGACAG GACT ACHV GGGT ATCT AATCC 3'	

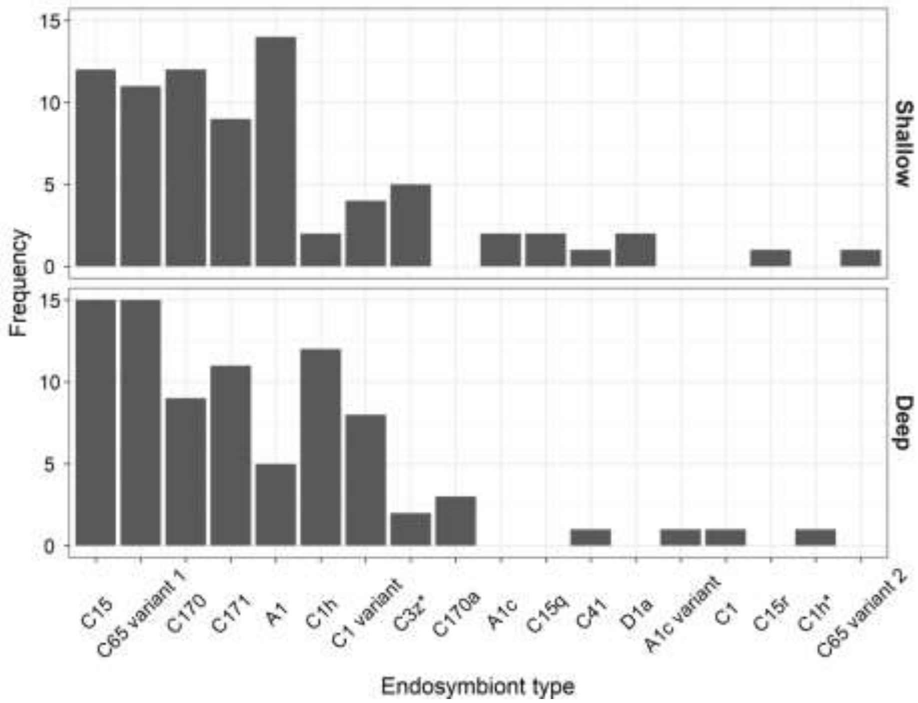

Figure S1. Bar blot shows the frequency distribution of endosymbiont ITS2 types collected from two depth levels (2-5 shallow and 15-18 deep) representing different light regime.

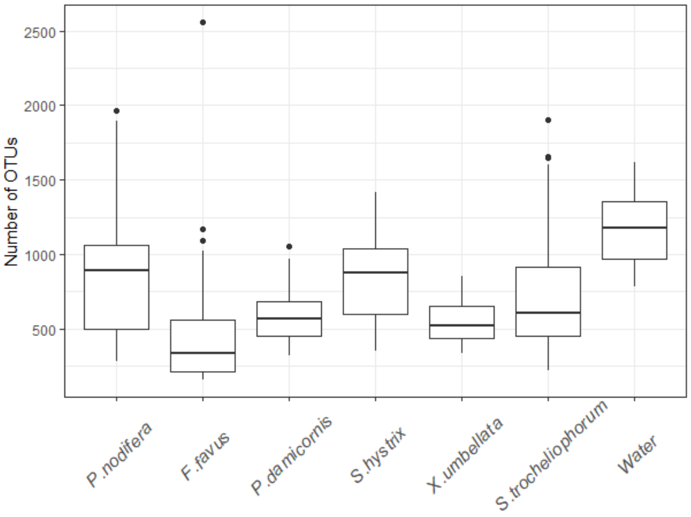

Figure 2S. Boxplot represents number of bacterial OTUs associated with coral mucus of six coral species obtained from two depth levels across five sites. The plot shows that median number of OTUs varied among species, but notably was higher in water samples.

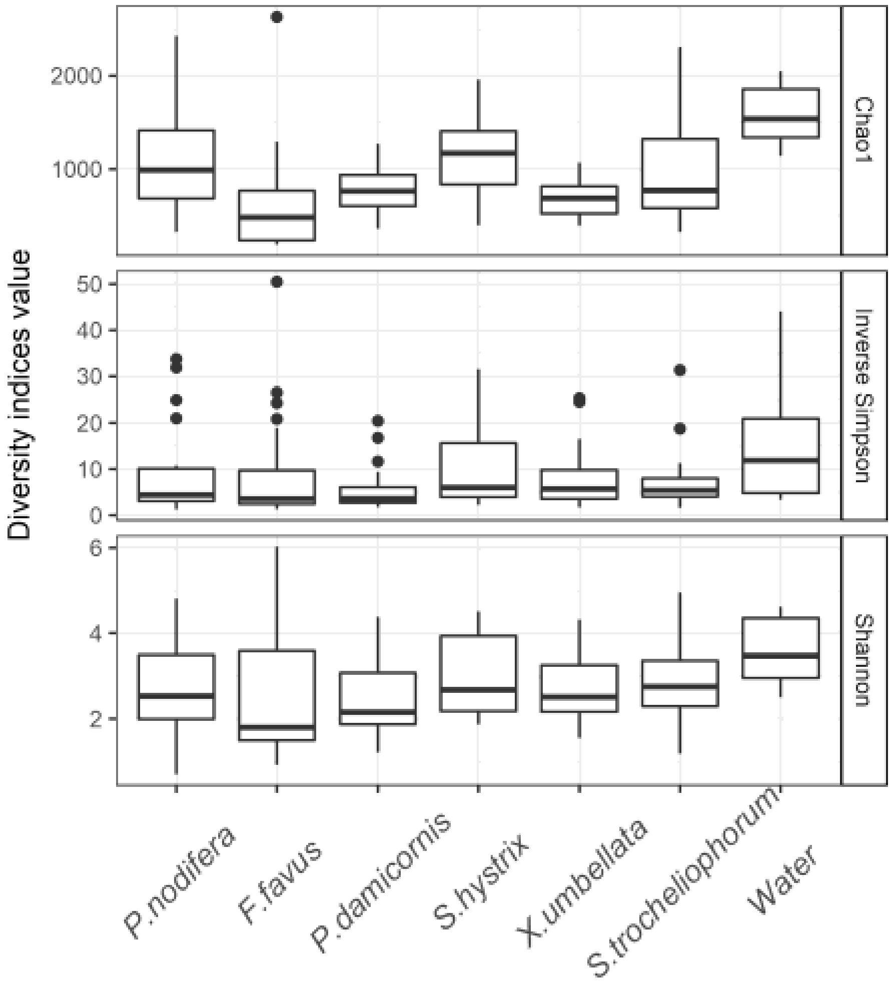

Figure S3. Box plots showing the richness and diversity indices values of mucus samples collected from six coral species at two levels along five surveyed sites in the northern Red Sea. The diversity of bacteria within water samples were higher than those associated with coral mucus.

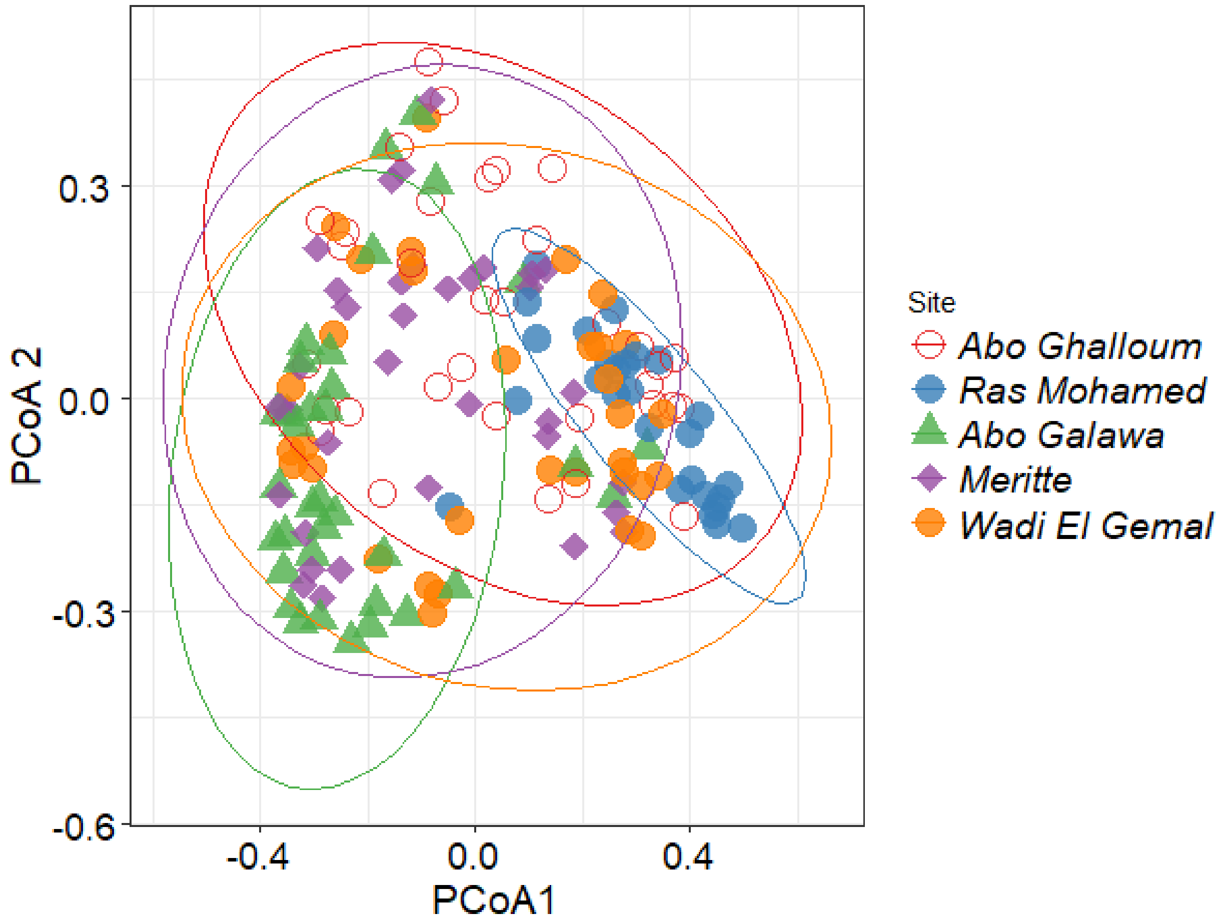

Figure S4. Principal Coordinate Analysis (PCoA) based on Bray-Curtis dissimilarity matrix of bacterial communities associated with six coral species at each site separately along thermal N-S gradient. Two most abundant OTUs (*Alteromonas* sp. and *Pseudoalteromonas* sp.) were not removed from this PCoA to show that their high relative abundances mask putative other patterns in the composition of other, less abundant bacterial OTUs.

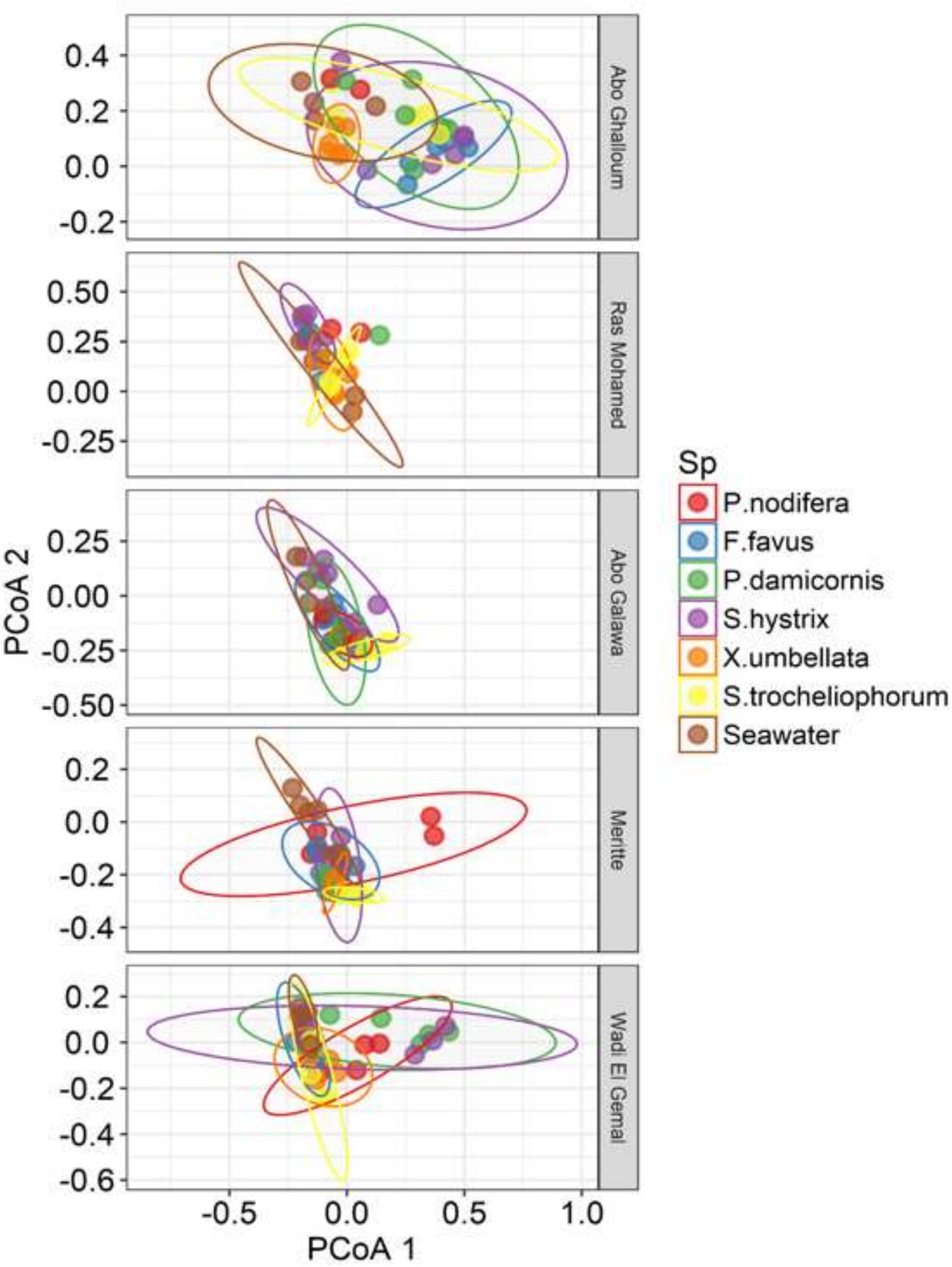

Figure S5. Principal Coordinate Analysis (PCoA) based on Bray-Curtis dissimilarity matrix of bacterial communities associated with six coral species at each site separately along thermal N-S gradient. Two most abundant OTUs (*Alteromonas* sp. and *Pseudoalteromonas* sp.) were found to mask geographic patterns and were therefore excluded for this visualization. The graph highlights the cluster of bacterial communities associated with each coral species separately suggesting species- specific bacteria within each site.

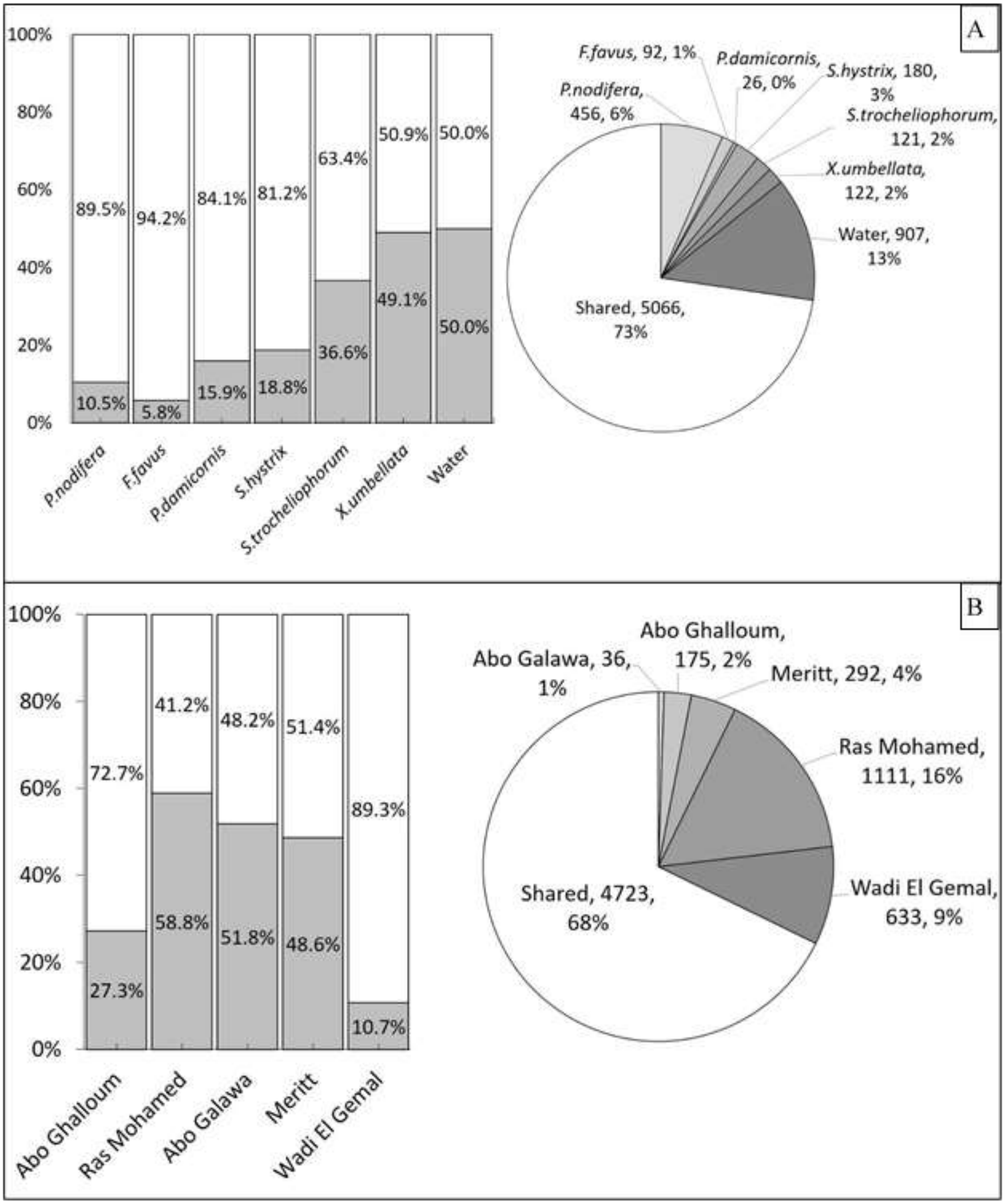

Figure S6. Indicator species analysis and the microbial community that was significantly ($p<0.05$) associated with the mucus collected from six coral species and five sampling sites, using Indi-species package in 'R'. Due to high similarity of the microbial community between depth levels, data were combined. Pie charts represent the number (and percentage) of OTUs that was significantly associated with each coral species (A) and sites (B), while barplots represent the contribution of those OTUs relative to total microbial abundance within each site and host.

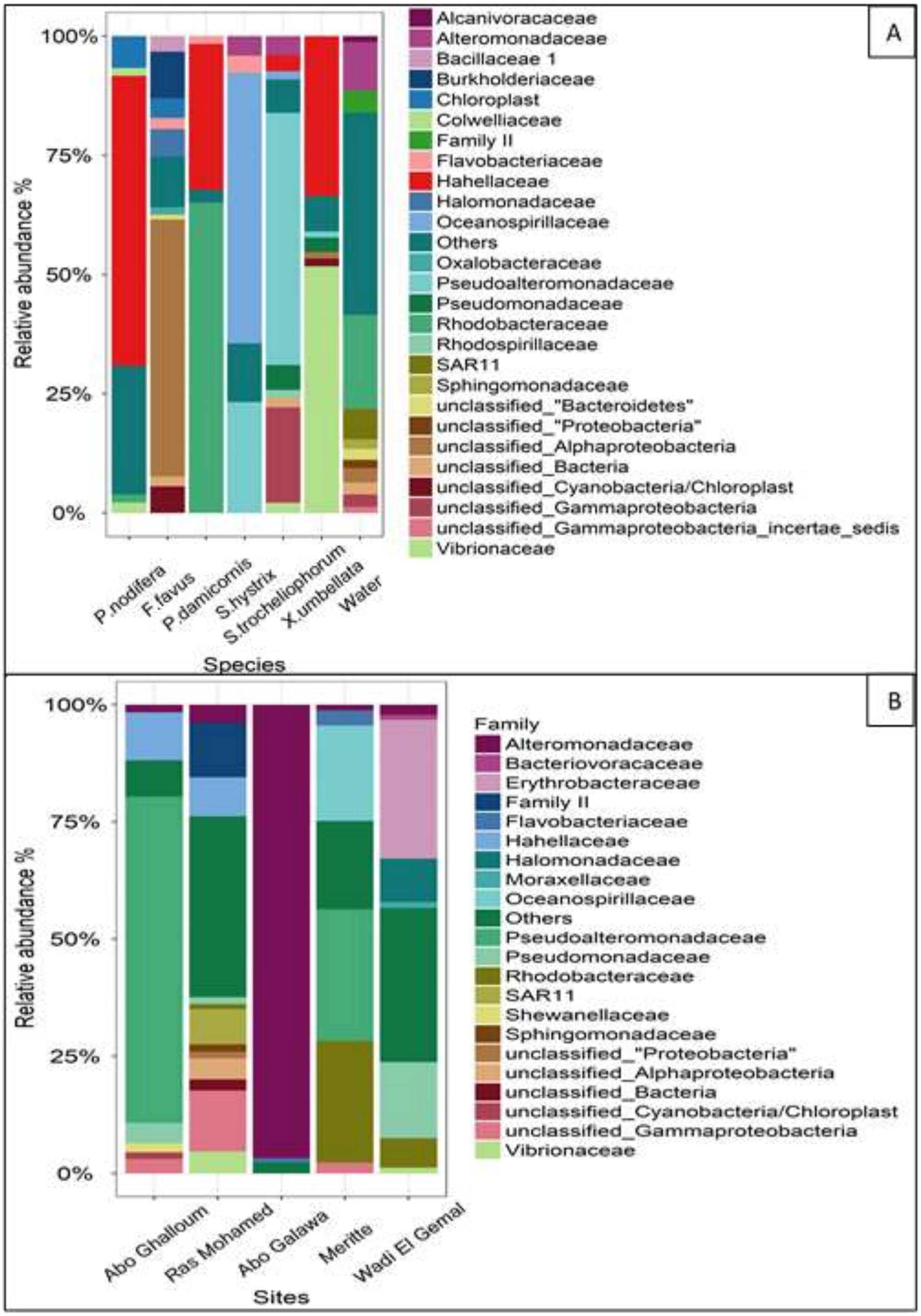

Figure S7. Taxonomic profile at family level of indicator species of microbial community that significantly ($p<0.05$) associated with each coral species (A) and sampling site (B) using Indi-species package in 'R'. Bar graph represent relative abundance of indicator bacteria at each site which clearly demonstrates that variation of taxonomy among sites and corals species despite the relatively high abundance of *Alteromonas* and *Pseudoalteromonas*. This highlights heterogeneity of bacterial community associated with sites across thermal gradient as well as species-specific bacteria associated with corals hosts.

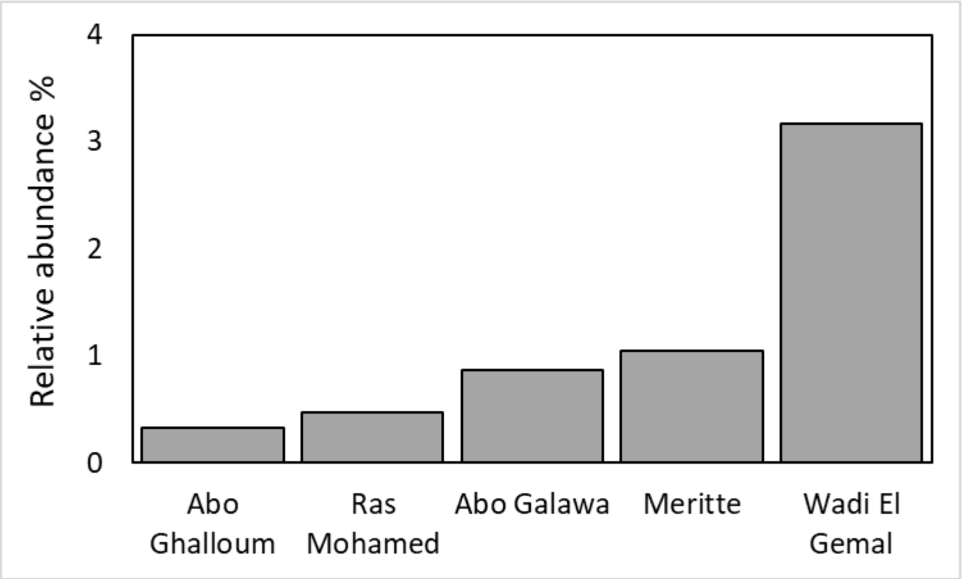

Figure S8. Relative abundance of photosynthetic *Erythrobacter* sp. (OTU10) that; i) comprised 1.2% of total bacterial abundance, ii) was the abundant indicator species in the warmest site (Wadi El Gemal), and iii) a core mucus bacterial OTU associated with 95% of all samples. This plot highlights the gradual increase in relative abundance of *Erythrobacter* across the thermal N-S gradient from the least warm site in the North (Abo Ghalloum) to the warmest site in the South (Wadi El Gemal) of the Northern Red Sea.

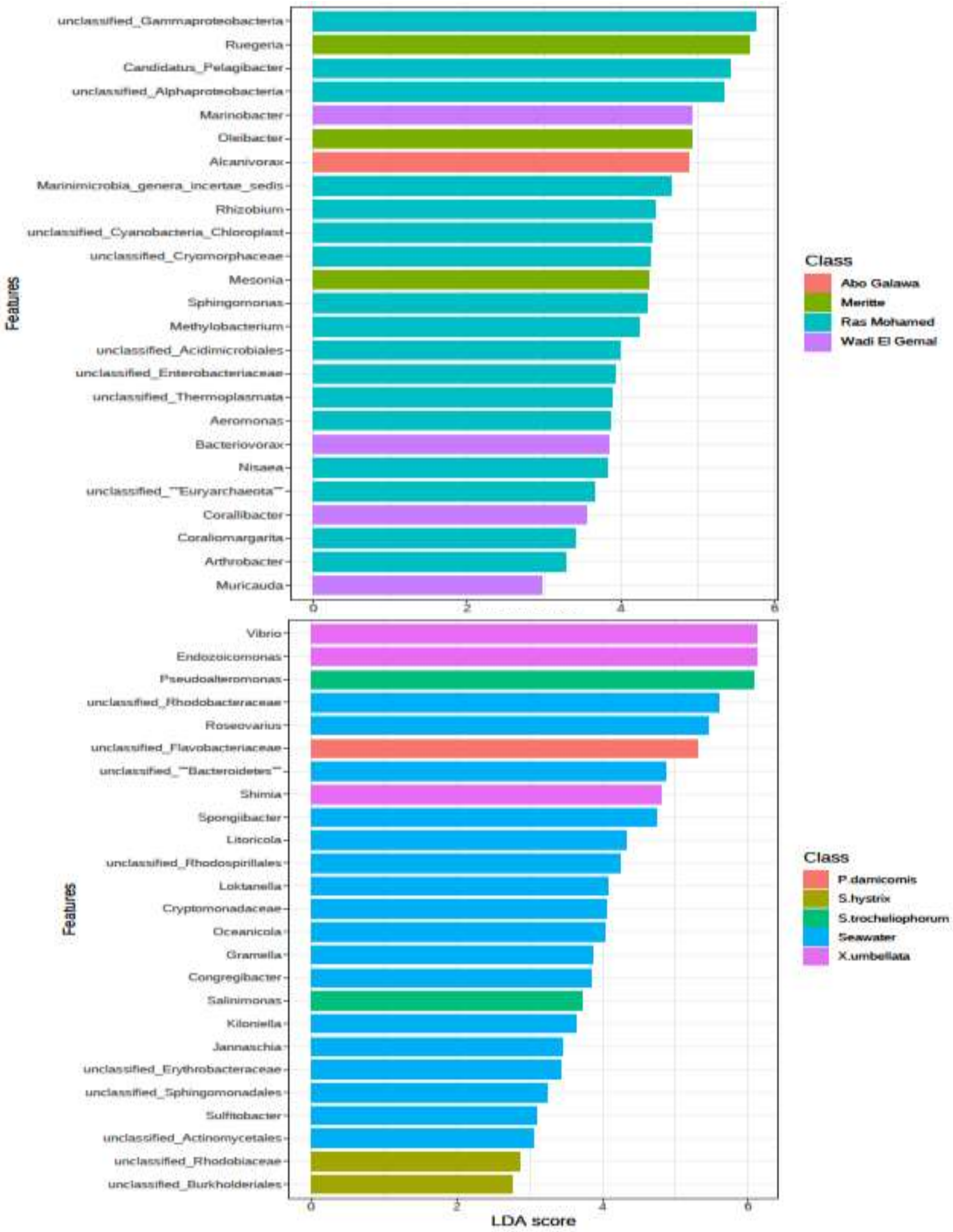

Figure S9. LEfSe analysis based on OTUs characterizing bacterial microbiomes between different sites and corals species. The bar graphs show the most significant OTUs (top 25 OTUs) that exhibit differential relative abundances between sites (a) and coral species (b).

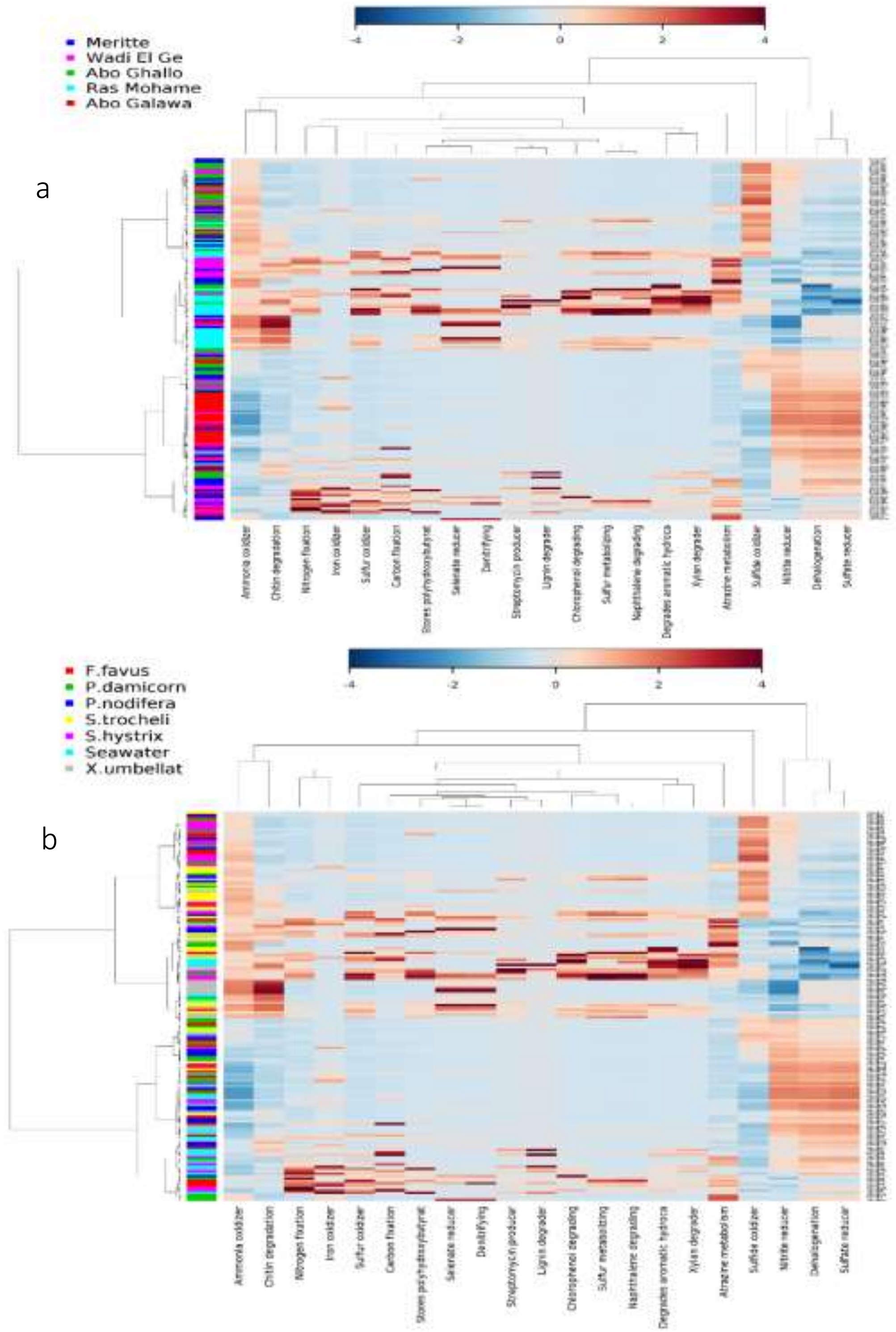

Figure S10. Taxonomy-based functional profiling of bacterial communities associated with SML of six coral species collected from five sites along latitudinal gradient of the northern Red Sea. The heatmap displays putative changes in bacterial community function between sites (a) and coral species (b). Changes in functional profiles between samples are displayed on a relative blue-red scale, while left bar shows the clustering pattern between sits and species. We used METAGENassist web portal (http://www.metagenassist.ca) with the default settings.

Analysis of Chlorophyll-*a* and light attenuation coefficient (K_d) as proxy for water quality and light stress along the study sites in the Red Sea

Monthly Chlorophyll-*a* content (Chl-*a*, mg/m^3) and Light Attenuation specific to 490nm (K_d, m^{-1}) were derived for five sites along the Red Sea (see osman et al., [1]) for the period 2003-2012 using Giovanni Ocean Colour tool; (http://giovanni.gsfc.nasa.gov/giovanni/), from the Moderate-resolution Imaging Spectroradiometer (MODIS) Aqua satellite (4 km resolution). Values for Chl-*a* and K_d for each month (m) were averaged across all years to estimate the Climatology (X_m) [equation 1], or monthly long term mean (± standard deviation), to subsequently calculate the monthly anomaly (A_m) and annual anomalies (A_a) as positive and negative deviations from their climatology (monthly long term mean) according to Maina *et al.*[2]as in equations 2 and 3.

$$X_m = \frac{\sum_{2012}^{2003} m}{ny}$$

[Equation 1]

Xm= average month value (*Jan 2003, Jan 2004,*…etc.), *ny*= number of studied years (10 years for Chl-*a*& K_d)

$$A_m = (X_m - Y_m)$$

[Equation **Error! No text of**

Ym= monthly value for each year (Jan 2003, Feb 2004,…etc.)

$$A_a = \frac{\sum_{Dec}^{Jan} A_m}{nm}$$

[Equation 3]

nm= number of months (12 months).

Differences of Chl-*a* and K_d between sites were evaluated using one-way ANOVAs after normality check (Shapiro test) and log transformation when necessary, and Tukey post-hoc test was used to identify significant differences across sites ($p<0.05$). Also, annual mean of environmental variables (SST, Chl-*a* and K_d combined) for the period 2003-2012 were used to perform Principal Component Analysis (PCA) to identify the the major environmental factor that drive the variability across sites.

Data revealed that Chl-*a* and K_d differed across sites, however the main source of variation was the south of the Northern Red Sea (i.e., Farasan - Fig. S8)). Mean values for Chl-*a* and K_d (2003–2012) were highest for the southernmost site (Farasan; 1.44 ± 0.26 mg/m^3 and 0.13 ± 0.02 m^{-1}) and generally declined northward and were lowest for the Gulf of Aqaba (0.19±0.06 mg/m^3 and 0.04±0.01 m^{-1}) (Fig. S8). Whilst the Gulf of Aqaba exhibited similar Chl-*a* and K_d regimes as Hurghada and Wadi El Gemal (Tukey's $p>0.05$) throughout this time frame, it exhibited clearer/less productive water (Fig. S8). Further, PCA analysis of Chl-*a*, K_d (MODIS-

Aqua) together with SST (CoRTAD-V5) based on the annual means across the period from 2003-2012 (Fig. S9) confirmed that SST was the major source of variation between sites along the Red Sea (PC1, 88% of variation), whereas Chl-*a* and K_d (PC2) together explained only 11.7% of variation (Fig. S9). From this, sites were clustered as Gulf of Aqaba, Hurghada and Wadi El Gemal together influanced by SST, unlike Farasan that was less influenced by SST and more from Chl-*a* and K_d (Fig. S9).

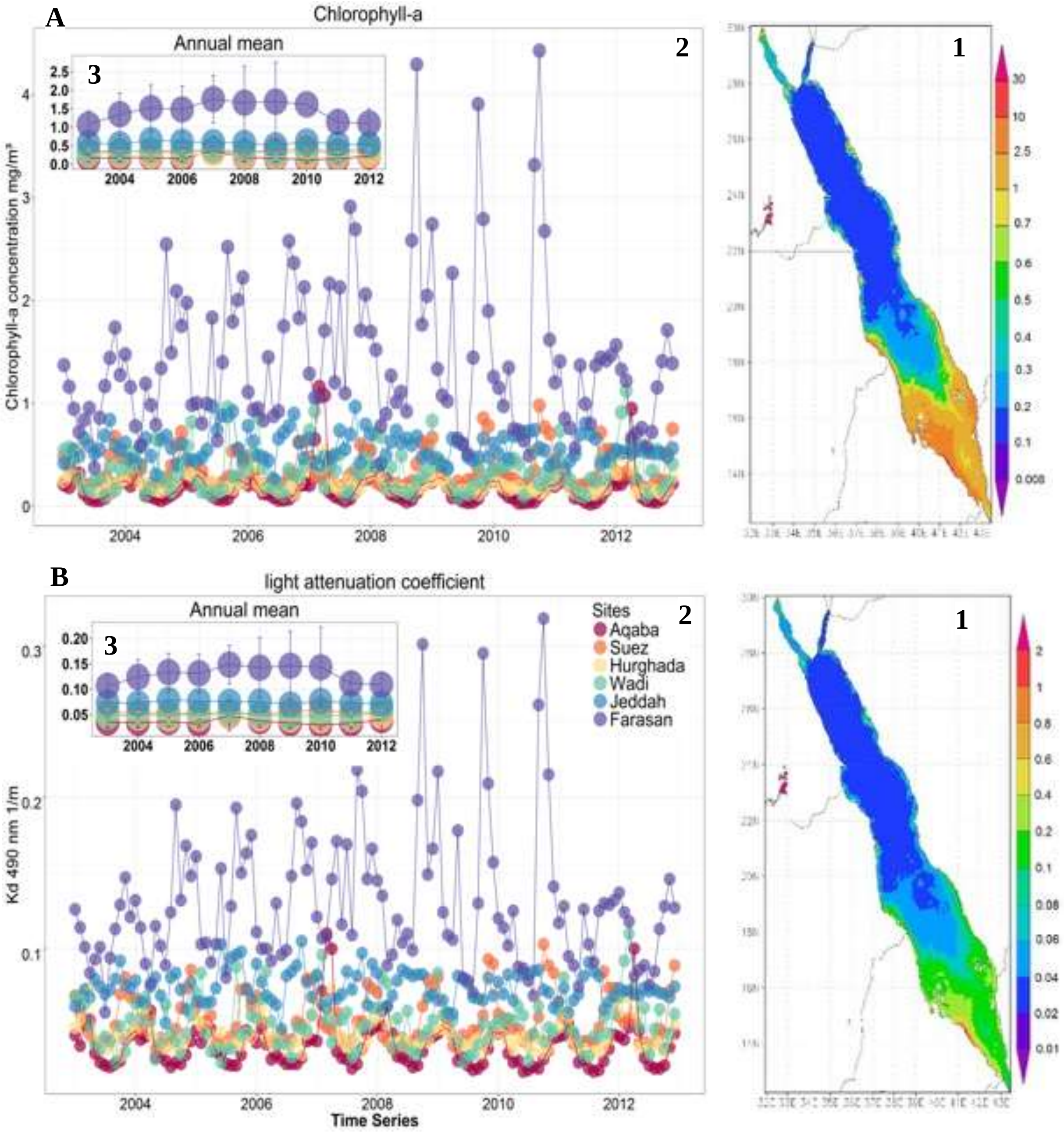

Figure S11. Colour-coded maps (left) produced by Giovanni ocean colour online tool during the period 2003 to 2012 and data obtained from remote sensing (MODIS, AQUA satellite 4 km). Maps show high values of Chlorophyll-*a* concentration (A-1) and K_d 490 nm (B-1) in south Red Sea influenced by intrusion of water from the Gulf of Aden. Monthly values (n=120) for Chl-*a* (A-2) and K_d (B-2) showed higher values in the Farasan region with high fluctuation values due to water exchange with Gulf of Aden. High SD of annual mean (n=12) as shown in A.3 & B.3.Data demonstrate the capability of coral in southern Red Sea to live in an environment with high fluctuation of Chl-*a* and K_d.

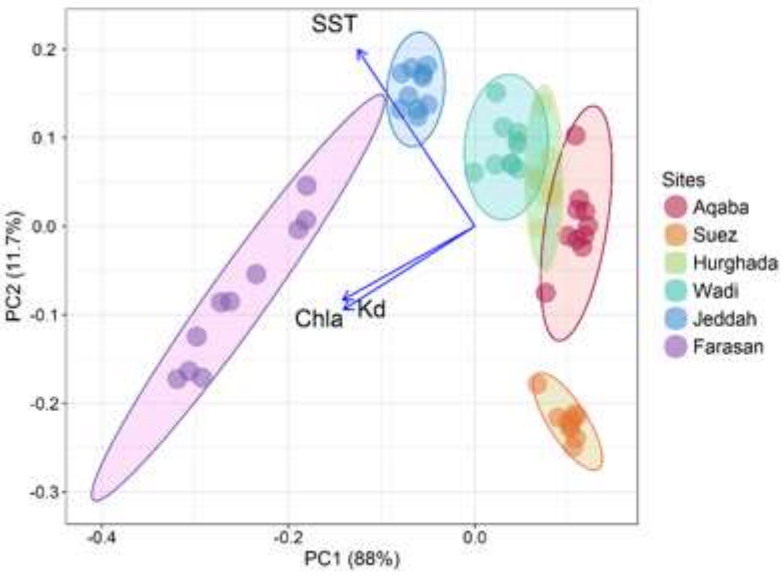

Figure S12 Principal component ordination (PCA) of environmental variables (SST, Chlorophyll-*a* and K_d 490 nm). Data clustered and color-coded by sites and each site represented by annual mean since 2003 to 2012. The major source of variation was PC1 (SST-88%) while PC2 (Chl-*a*) composed only 11.7% of the variation. The direction of loading for each parameter vector is indicated (blue arrows).

Measurement of ammonia concentration

Seawater samples from each site were collected to measure (in the laboratory) the variability of ammonia, phosphate and nitrate concentration between sampling sites. Due to some difficulties (logistics and mishap in the laboratory), unfortunately we were able to measure only ammonia concentration and therefore we did not include it in the study. Briefly, three water samples replicates were collected at each site (n=3) at each depth in 500 ml polyethylene bottle during the sampling time to measure ammonia concentrations across five sites. Seawater samples were preserved in the fridge at 4°C straight after sampling and then transported to Marine Biology lab, Al Azhar University, Egypt, for analysis. Analysis of seawater samples were carried out using standard protocol as per APHA (1998) protocol using titrations and colorimetric detection of ammonia. To analyze ammonia concentration between sites, data normality was checked, and One-Way ANOVA was performed on R. Data showed that mean of ammonia concentration ranged from 1.26±0.7 in Ras Mohamed to 2.9±0.8 µg/L at Abo Galawa (Fig. S10) without significant difference between sites (ANOVA, $F_{4,15}=1.6$, $p=0.25$). The invariability of

ammonia concentration combined with remote sensing data strongly suggests non-significant changes in seawater parameters (temperature excluded) between study sites along the selected N-S gradient, which are not likely to drive the difference of bacterial community structure.

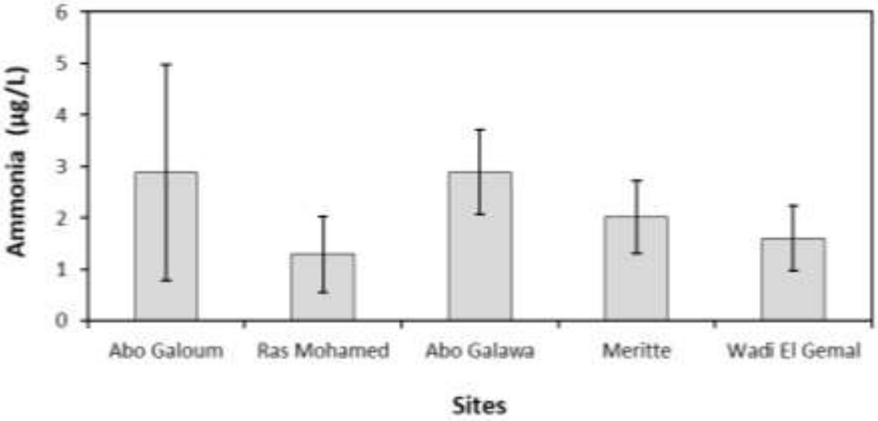

Figure S13. Seawater ammonia concentration at each study site along the N-S gradient in the northern Red Sea.

References

1. Osman EO, Smith DJ, Ziegler M, Kürten B, Conrad C, El-Haddad KM, et al. Thermal refugia against coral bleaching throughout the northern Red Sea. Glob Chang Biol. 2017; August:1–11. doi:10.1111/gcb.13895.

2. Maina J, Venus V, McClanahan TR, Ateweberhan M. Modelling susceptibility of coral reefs to environmental stress using remote sensing data and GIS models. Ecol Modell. 2008;212:180–99. doi:10.1016/j.ecolmodel.2007.10.033.
