# Supplementary material for: Coral microbiome composition along the northern Red Sea suggests high plasticity of bacterial and specificity of endosymbiotic dinoflagellate communities
Source: Microbiome. 2020 Feb 6;8:8. doi: 10.1186/s40168-019-0776-5 (PMC6996193; doi:10.1186/s40168-019-0776-5)
Supplement: Supplementary file 5 — Additional file 5. Normalization and-beta-diversity. [file 40168_2019_776_MOESM5_ESM.pdf]

Beta diversity

EO

```
require(readxl)

## Loading required package: readxl

df <- read_excel("D:/My PhD/papers/Microbiom paper/Microbiom paper/New OTU ta
ble/Data/Matrix_for_analysis_metadata.xlsx")

mat=df[,12:6981]
meta=df[,1:11]
```

This is the rarefaction curve of the non-normalized dataset. Plot shows low variance in sequence depth between samples (except one outlier).

```
rarecurve(mat)
```

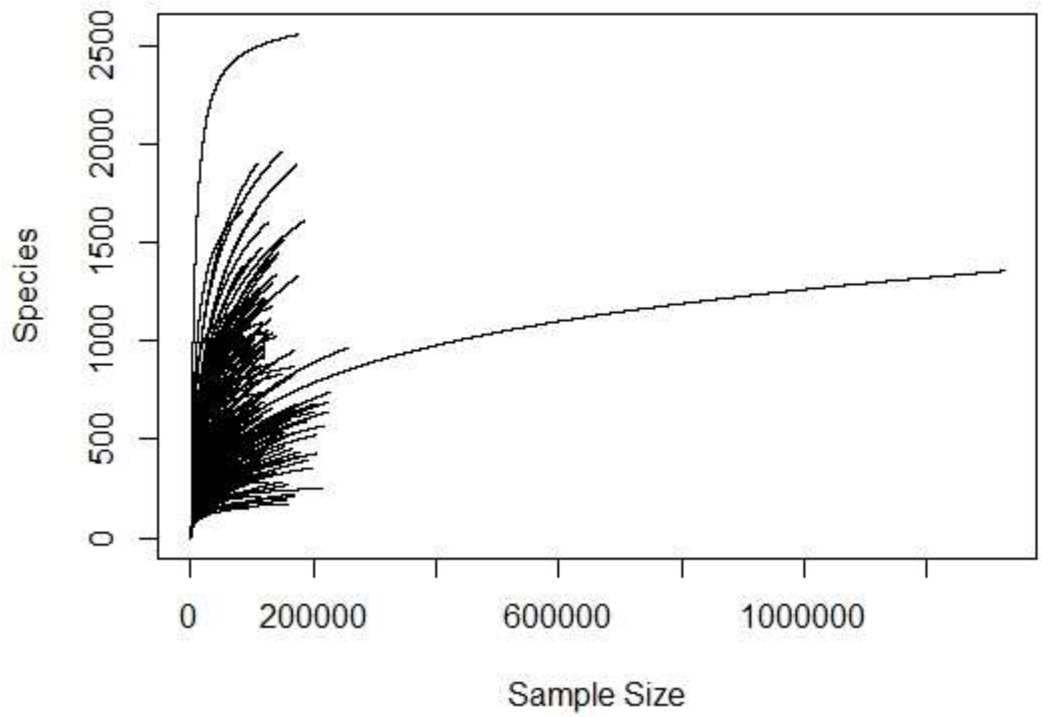

#Rarefaction to minimum value

Let's get a sense of sequence depth variance first

```
depth=rowSums(mat)# to calculate the samples seq depth
hist(depth) # plot seq depth it as histogram
```

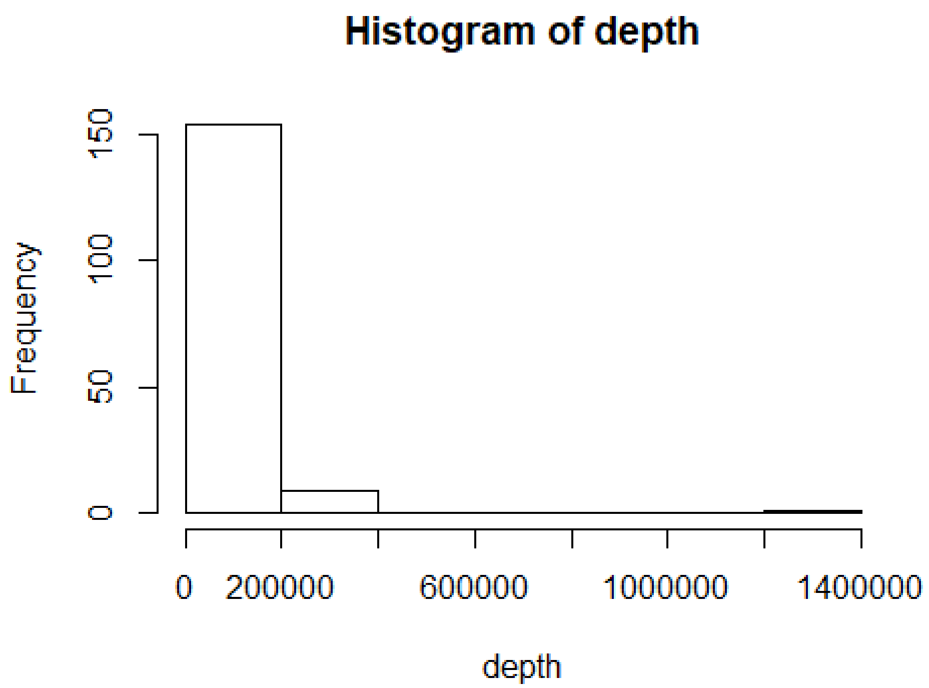

This graph highlights that most of our samples fallen under same data sequance range with few outliers.

Let's rarify our samples anyway to the minimum value of sequence depth:

```
require(vegan)

## Loading required package: vegan

## Loading required package: permute

## Loading required package: lattice

## This is vegan 2.5-5

rare_mat=rrarefy(mat, sample = min(depth)) # the new data frame is rare_mat w
hich will be used later in the analysis
```

Let's check Beta diversity for rarified data matrix:

```r
# to calculate the dissimilarity distance using Bray method
dist_rare=vegdist(rare_mat, "bray")

# To calculate PCoA values from dissimilarity
PCoAValue_rare=wcmdscale(dist_rare, k=2)

# to transfare it to data frame
PCoAValue_rare=as.data.frame(PCoAValue_rare)

# To import the meta data
PCoAValue_rare$Sp=meta$Sp
PCoAValue_rare$Site=meta$Site
PCoAValue_rare$Depth=meta$Depth


# To arrange our sites in latitudianl order
PCoAValue_rare$Site=factor(PCoAValue_rare$Site, levels = c("Abo Galloum", "Ra
s Mohamed", "Abo Galawa", "Meritte", "Wadi ElGemal"))

PCoAValue_rare$Sp=factor(PCoAValue_rare$Sp,
                    levels = c("P.nodifera",
                               "F.favus",
                               "P.damicornis",
                               "S.hystrix",
                               "X.umbellata",
                               "S.trocheliophorum",
                               "Water"))



#To plot the PCoA - NOTE that we used here rarified data (PCoAValue_rare)
require(ggplot2)

## Loading required package: ggplot2

ggplot(PCoAValue_rare, aes(x= V1, y= V2, color=Site, shape=Site))+
  geom_point(size=5, alpha=0.8)+
  scale_shape_manual(values=c(1,15, 16, 17,18,19,20))+
  theme_bw()+
  scale_color_brewer(palette = "Set1")+
  labs(x="PCoA1",y="PCoA 2")+
  theme(
    axis.text.x=
      element_text(
        color = "black",
        size=12,
        angle=0,
        hjust=.5,
        vjust=.5,
```

```r
      face="plain"))+
theme(
  axis.text.y =
    element_text(
      colour="black",
      size=12,
      angle=0,
      hjust=.5,
      vjust=.5,
      face="plain"))+
theme(
  axis.title.x =
    element_text(
      face="plain",
      size=14,
      vjust=-1,
      angle = 0))+
theme(
  axis.title.y =
    element_text(
      face="plain",
      size=14,
      vjust=1.5))+
theme(
  legend.text =
    element_text(
      colour="black",
      size = 12,
      face = "italic"))+
theme(
  legend.title =
    element_text(
      colour="black",
      size=12,
      face="plain"))+
theme(strip.text.x =
        element_text(
          size = 12), strip.background=element_blank())+
stat_ellipse()
```

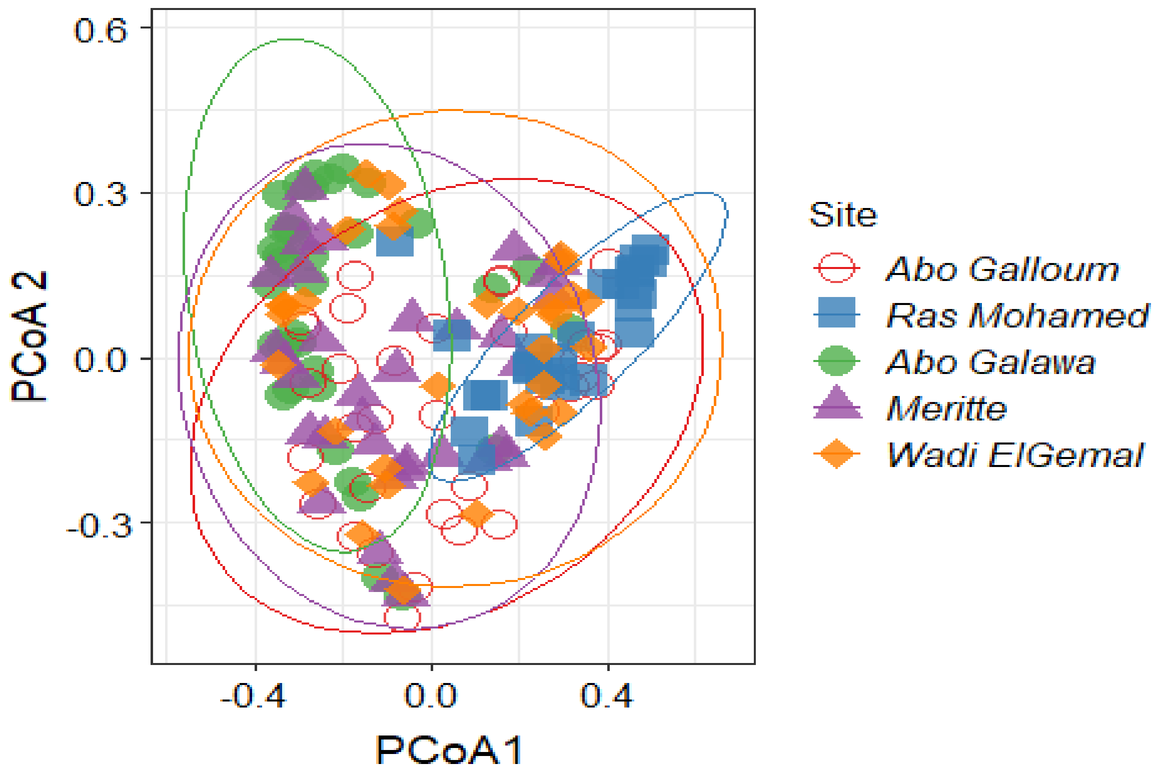

We here visualize the 16S rRNA data without removal of the two most abundant OTUs. It is obvious that it is hard to discern any patterns in the dataset. Let's go and have a look at the PCoA after removal of the most two abundant OTUs as in the MS.

First, our data is arranged in a declining order of relative OTU abundance. The first two columns are most two abundant OTUs, so we will removed first two columns.

Sanity check first

```
OTU_abun=colSums(mat)
head (OTU_abun, n=20)

## OTU1;size=1888331;  OTU2;size=654207;  OTU94;size=14521;
##            5851880            2208755            997177
##  OTU4;size=344109;  OTU15;size=70952;  OTU29;size=38572;
##             722904             470821            467620
##  OTU6;size=192385;  OTU14;size=79553;  OTU7;size=134453;
##             400646             378498            298855
##  OTU8586;size=140; OTU10;size=102055;  OTU32;size=37105;
##             252966             252405            236331
##  OTU11;size=98019;  OTU80;size=17615;  OTU28;size=38788;
##             226023             221409            193932
##  OTU17;size=61389;  OTU12;size=96996;  OTU22;size=52572;
##             191452             190784            170746
##  OTU20;size=57666;  OTU32233;size=38;
##             167536             164892
```

```
mat_minus_2OTUs=mat[,-c(1:2)] #to remove first two columns, so the new matrix
is called "mat_minus_2OTUs"
```

Let's plot the PCoA - Please note again that we removed the most abundant OTUs only for this plot only. All statistical analysis were done on complete dataset **without removal of those OTUs.**

```
# to calculate the dissimilarity distance using Bray method
dist_minus2OTUs=vegdist(mat_minus_2OTUs, "bray")

# To calculate PCoA values from dissimilarity
PCoAValue_minus2OTUs=wcmdscale(dist_minus2OTUs, k=2)

# to transfare it to data frame
PCoAValue_minus2OTUs=as.data.frame(PCoAValue_minus2OTUs)

# To import the meta data
PCoAValue_minus2OTUs$Sp=meta$Sp
PCoAValue_minus2OTUs$Site=meta$Site
PCoAValue_minus2OTUs$Depth=meta$Depth



# To arrange our sites in latitudianl order
PCoAValue_minus2OTUs$Site=factor(PCoAValue_minus2OTUs$Site, levels = c("Abo G
alloum", "Ras Mohamed", "Abo Galawa", "Meritte", "Wadi ElGemal"))

PCoAValue_minus2OTUs$Sp=factor(PCoAValue_minus2OTUs$Sp,
                    levels = c("P.nodifera",
                               "F.favus",
                               "P.damicornis",
                               "S.hystrix",
                               "X.umbellata",
                               "S.trocheliophorum",
                               "Water"))




#To plot the PCoA - NOTE that we used here rarified data (PCoAValue_minus2OTU
s)

ggplot(PCoAValue_minus2OTUs, aes(x= V1, y= V2, color=Site, shape=Site))+
  geom_point(size=5, alpha=0.8)+
  scale_shape_manual(values=c(1,15, 16, 17,18,19,20))+
  theme_bw()+
  scale_color_brewer(palette = "Set1")+
  labs(x="PCoA1",y="PCoA 2")+
  theme(
    axis.text.x=
      element_text(
```

```
      color = "black",
      size=12,
      angle=0,
      hjust=.5,
      vjust=.5,
      face="plain"))+
theme(
  axis.text.y =
    element_text(
      colour="black",
      size=12,
      angle=0,
      hjust=.5,
      vjust=.5,
      face="plain"))+
theme(
  axis.title.x =
    element_text(
      face="plain",
      size=14,
      vjust=-1,
      angle = 0))+
theme(
  axis.title.y =
    element_text(
      face="plain",
      size=14,
      vjust=1.5))+
theme(
  legend.text =
    element_text(
      colour="black",
      size = 12,
      face = "italic"))+
theme(
  legend.title =
    element_text(
      colour="black",
      size=12,
      face="plain"))+
theme(strip.text.x =
        element_text(
          size = 12), strip.background=element_blank())+
stat_ellipse()
```

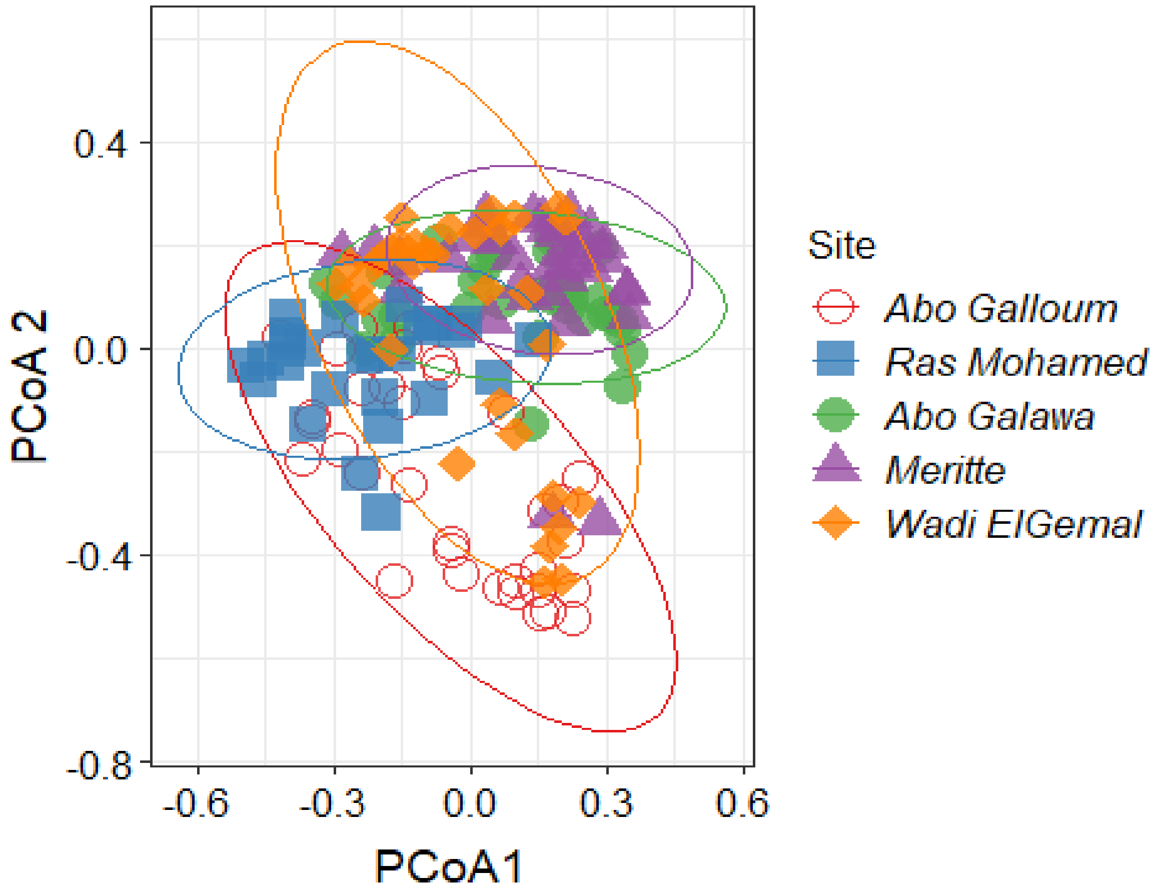

This looks exactly the same as Figure 3 in our MS. So, rarefaction did not change the beta diversity.

To do PERMANOVA on rarified matrix:

```
adonis(rare_mat ~ Sp+Site+Depth, data=meta, permutations=9999)

##
## Call:
## adonis(formula = rare_mat ~ Sp + Site + Depth, data = meta, permutations =
9999)
##
## Permutation: free
## Number of permutations: 9999
##
## Terms added sequentially (first to last)
##
##            Df SumsOfSqs MeanSqs F.Model      R2 Pr(>F)
## Sp          6     8.158 1.35960  6.8596 0.17566 0.0001 ***
## Site        4     7.843 1.96087  9.8931 0.16890 0.0001 ***
## Depth       1     0.311 0.31071  1.5676 0.00669 0.0906 .
## Residuals 152    30.127 0.19821         0.64875
## Total     163    46.439                 1.00000
```

```
## ---
## Signif. codes:  0 '***' 0.001 '**' 0.01 '*' 0.05 '.' 0.1 ' ' 1
```

These results shows that coral species and sites significantly influence the bacterial community, but not depth, as stated in the MS. Also, note the R2 value is quite similar to our data stated in the MS (Line 28-210).

Total Sum scaling normalization

```
Mat_perc=t(apply(mat, 1, function(x)(x)*100/(sum(x))))  # To normalize each s
ample to 100%
rowSums(Mat_perc)   # sanity check to see all samples equal 100
```

```
##   [1] 100 100 100 100 100 100 100 100 100 100 100 100 100 100 100 100 100
##  [18] 100 100 100 100 100 100 100 100 100 100 100 100 100 100 100 100 100
##  [35] 100 100 100 100 100 100 100 100 100 100 100 100 100 100 100 100 100
##  [52] 100 100 100 100 100 100 100 100 100 100 100 100 100 100 100 100 100
##  [69] 100 100 100 100 100 100 100 100 100 100 100 100 100 100 100 100 100
##  [86] 100 100 100 100 100 100 100 100 100 100 100 100 100 100 100 100 100
## [103] 100 100 100 100 100 100 100 100 100 100 100 100 100 100 100 100 100
## [120] 100 100 100 100 100 100 100 100 100 100 100 100 100 100 100 100 100
## [137] 100 100 100 100 100 100 100 100 100 100 100 100 100 100 100 100 100
## [154] 100 100 100 100 100 100 100 100 100 100 100
```

Let’s check Beta diversity:

```
# to calculate the dissimilarity distance using Bray method
dist_perc=vegdist(Mat_perc, "bray") # Note, here we used new TSS matrix

# To calculate PCoA values from dissimilarity
PCoAValue_perc=wcmdscale(dist_perc, k=2)

# to transfare it to data frame
PCoAValue_perc=as.data.frame(PCoAValue_perc)

# To import the meta data
PCoAValue_perc$Sp=meta$Sp
PCoAValue_perc$Site=meta$Site
PCoAValue_perc$Depth=meta$Depth



# To arrange our sites in latitudinal order
PCoAValue_perc$Site=factor(PCoAValue_perc$Site, levels = c("Abo Galloum", "Ra
s Mohamed", "Abo Galawa", "Meritte", "Wadi ElGemal"))

PCoAValue_perc$Sp=factor(PCoAValue_perc$Sp,
                     levels = c("P.nodifera",
                                "F.favus",
                                "P.damicornis",
```

```
                                    "S.hystrix",
                                    "X.umbellata",
                                    "S.trocheliophorum",
                                    "Water"))



#To plot the PCoA - NOTE that we used here rarified data (PCoAValue_perc)

ggplot(PCoAValue_perc, aes(x= V1, y= V2, color=Site, shape=Site))+
  geom_point(size=5, alpha=0.8)+
  scale_shape_manual(values=c(1,15, 16, 17,18,19,20))+
  theme_bw()+
  scale_color_brewer(palette = "Set1")+
  labs(x="PCoA1",y="PCoA 2")+
  theme(
    axis.text.x=
      element_text(
        color = "black",
        size=12,
        angle=0,
        hjust=.5,
        vjust=.5,
        face="plain"))+
  theme(
    axis.text.y =
      element_text(
        colour="black",
        size=12,
        angle=0,
        hjust=.5,
        vjust=.5,
        face="plain"))+
  theme(
    axis.title.x =
      element_text(
        face="plain",
        size=14,
        vjust=-1,
        angle = 0))+
  theme(
    axis.title.y =
      element_text(
        face="plain",
        size=14,
        vjust=1.5))+
  theme(
    legend.text =
      element_text(
```

```
        colour="black",
        size = 12,
        face = "italic"))+
  theme(
    legend.title =
      element_text(
        colour="black",
        size=12,
        face="plain"))+
  theme(strip.text.x =
          element_text(
            size = 12), strip.background=element_blank())+
  stat_ellipse()
```

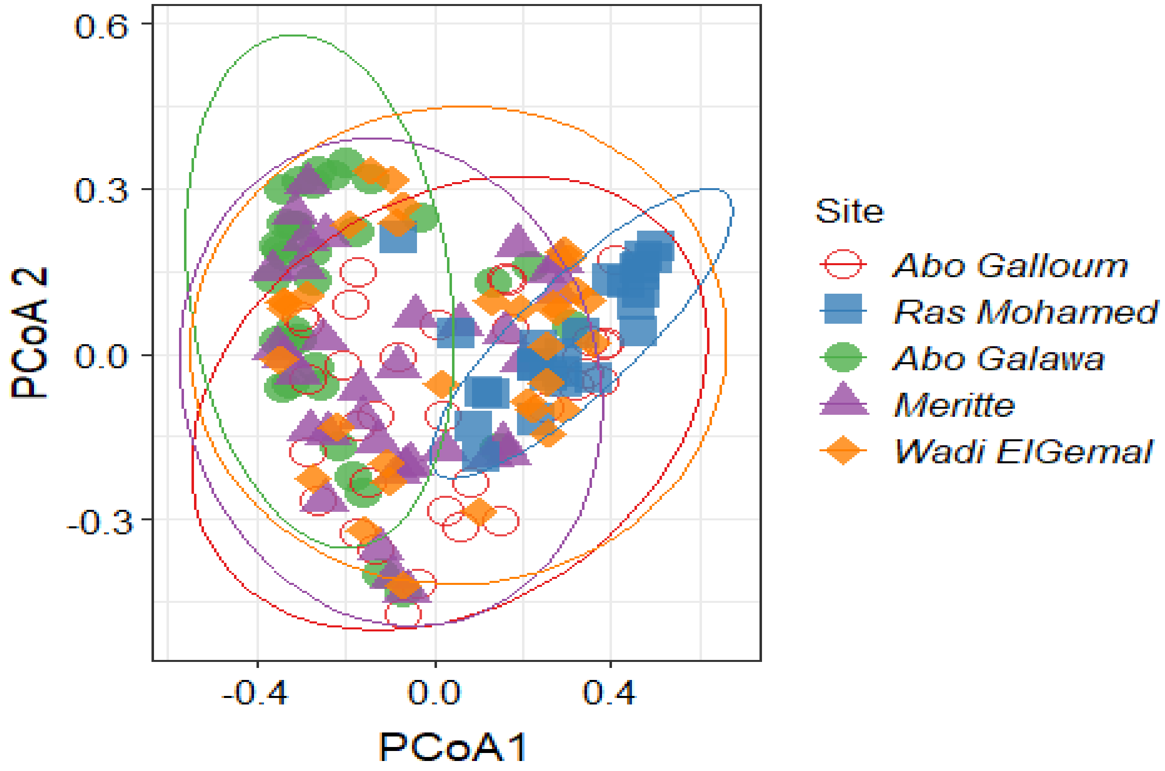

This is the same cluster pattern obtained as for rarefied samples.

This is to check PERMANOVA for the new SST normalized dataset:

```
adonis(Mat_perc ~ Sp+Site+Depth, data=meta, permutations=9999) # agian we use
the new SST normalized dataset

##
## Call:
## adonis(formula = Mat_perc ~ Sp + Site + Depth, data = meta, permutations =
9999)
##
## Permutation: free
```

```
## Number of permutations: 9999
##
## Terms added sequentially (first to last)
##
##            Df SumsOfSqs MeanSqs F.Model      R2 Pr(>F)
## Sp          6     8.166 1.36104  6.8901 0.17613 0.0001 ***
## Site        4     7.864 1.96600  9.9526 0.16961 0.0001 ***
## Depth       1     0.310 0.30974  1.5680 0.00668 0.1039
## Residuals 152    30.026 0.19754         0.64758
## Total     163    46.366                 1.00000
## ---
## Signif. codes:  0 '***' 0.001 '**' 0.01 '*' 0.05 '.' 0.1 ' ' 1
```

Again, we have the same conclusion where site and host species affected the bacterial community, but not depth. Further, similar R2 values were obtained as rarefied and non-rarefied datasets.

Conclusion

We therefore used non normalized approach as it does not change beta diversity.
