# Supplementary material for: Coral microbiome composition along the northern Red Sea suggests high plasticity of bacterial and specificity of endosymbiotic dinoflagellate communities
Source: Microbiome. 2020 Feb 6;8:8. doi: 10.1186/s40168-019-0776-5 (PMC6996193; doi:10.1186/s40168-019-0776-5)
Supplement: Supplementary file 6 — Additional file 6. Endoymbiont DGGE fingerprints. [file 40168_2019_776_MOESM6_ESM.pdf]

PCR-DGGE fingerprints of *Symbiodinium* community hosted within *Porites nodifera*, *Favia favus*, *Pocillopora damicornis*, *Seriatopora hystrix*, *Xenia umbellata* and *Sarcophyton trocheliophorum* collected from five sites at two depths (n=15 at each depth) along Egyptian Red Sea latitudes in Febuary-2013. The symbiont type (Clade ID) is given for each lane (sample) where letters indicate lineage type (clade) and numbers indicate ITS2 type (subclade) according to gene bank database as well as to Dr. Eugenia Sampayo (Uni of Queensland, AU) and Prof. Todd LaJeunesse (Penn. State University, US) collection. Arrows describe the characteristics bands of the fingerprint and example of heteroduplexes are also indicated.

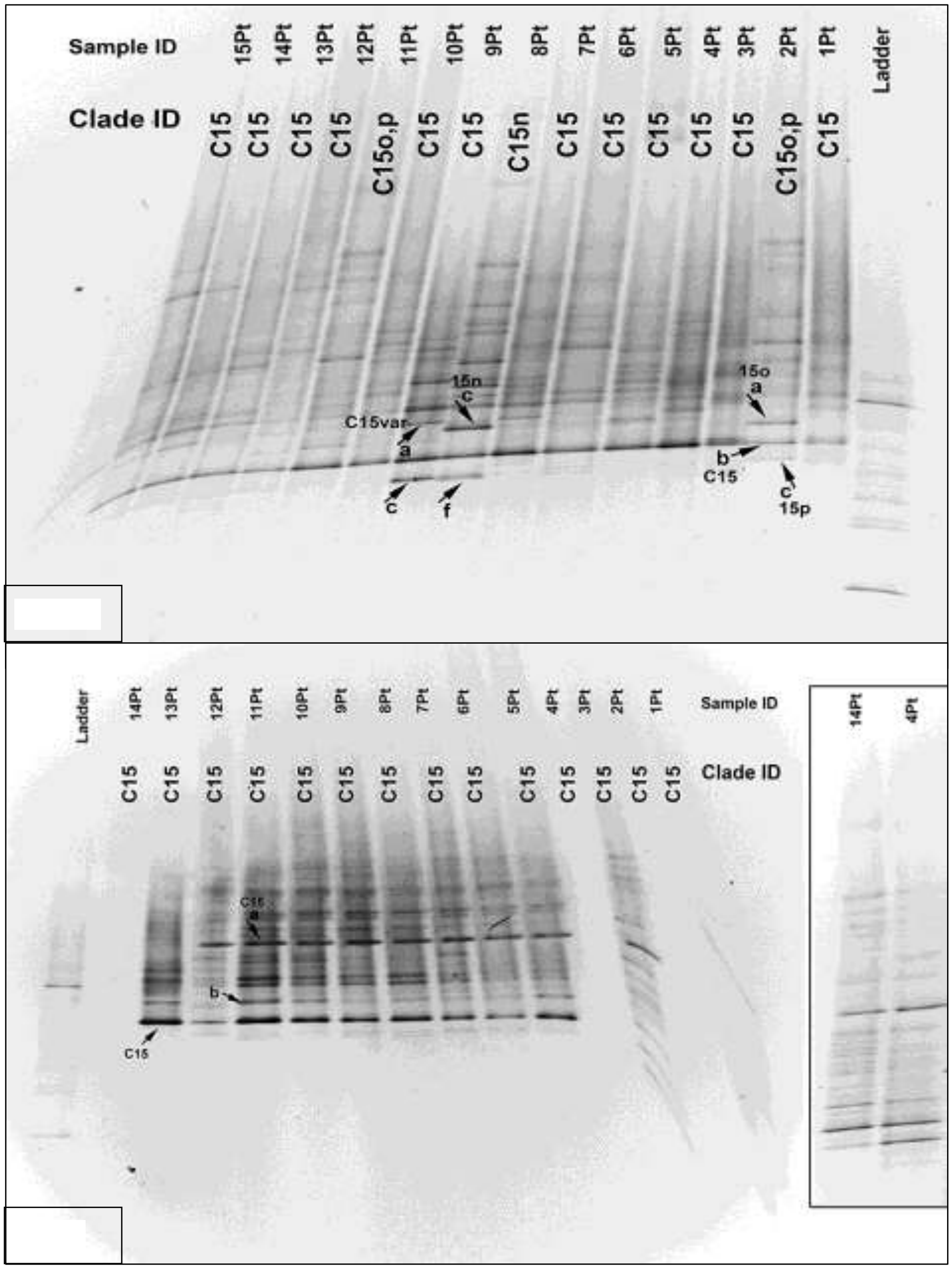

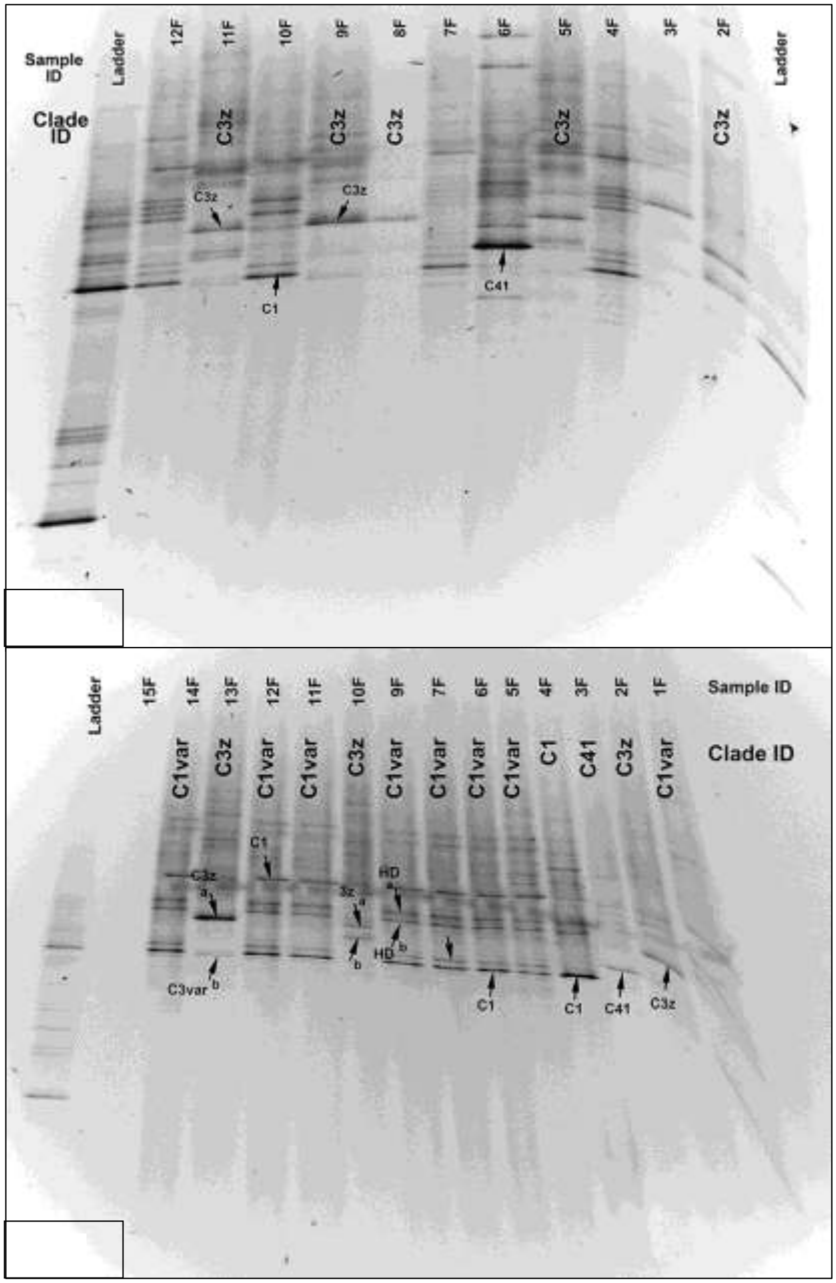

Sample ID
Clade ID
Ladder
12F
11F
10F
9F
8F
7F
6F
5F
4F
3F
2F
Ladder
C3z
C3z
C3z
C3z
C3z
C3z
C3z
C1
C41
Ladder
15F
14F
13F
12F
11F
10F
9F
7F
6F
5F
4F
3F
2F
1F
Sample ID
C1var
C3z
C1var
C1var
C3z
C1var
C1var
C1var
C1var
C1
C41
C3z
C1var
Clade ID
C3z
a
C3var
b
C1
3z
a
b
HD
a
HD
b
C1
C1
C41
C3z

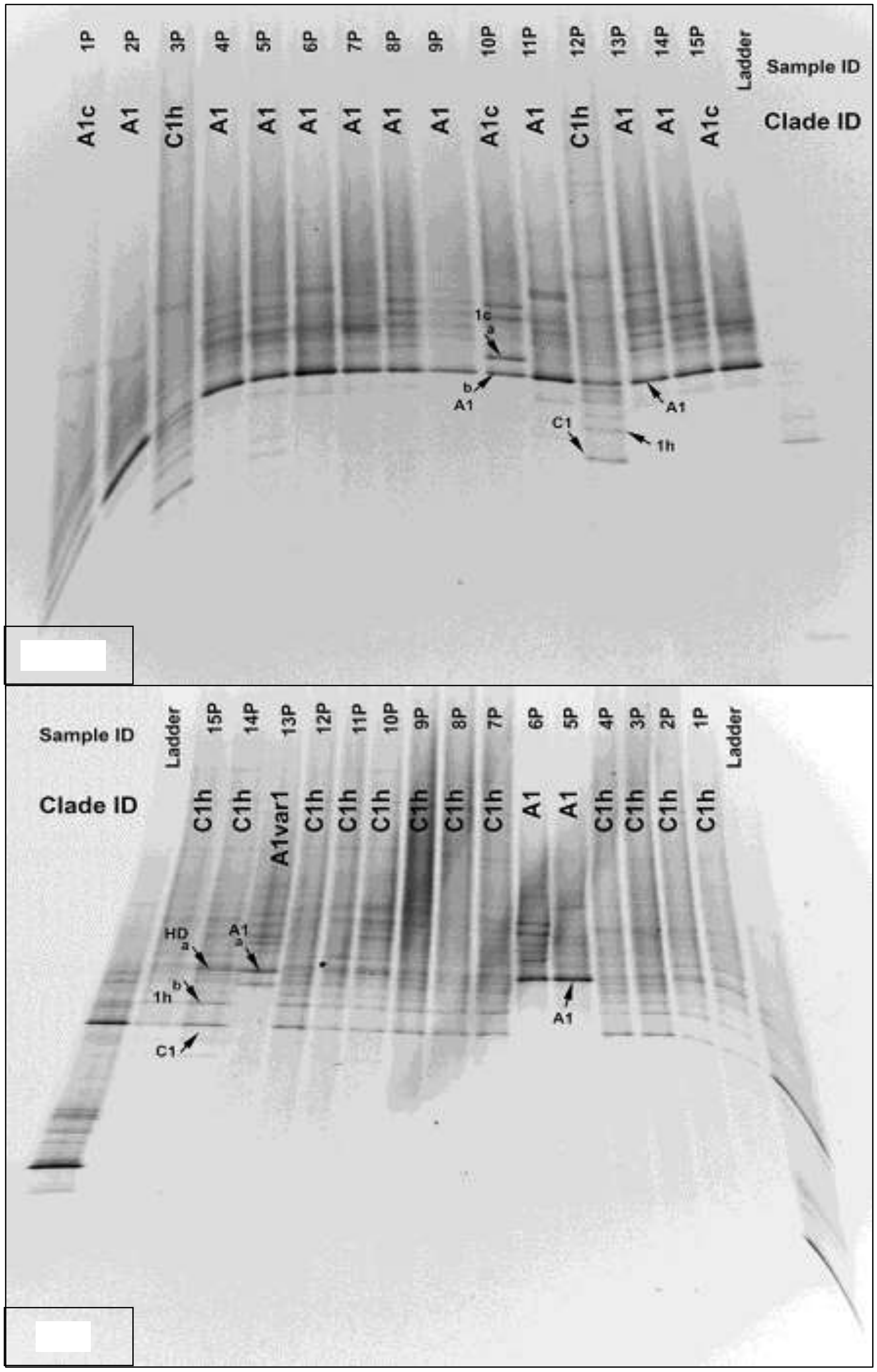

Sample ID
Clade ID
1P 2P 3P 4P 5P 6P 7P 8P 9P 10P 11P 12P 13P 14P 15P Ladder
A1c A1 C1h A1 A1 A1 A1 A1 A1 A1c A1 C1h A1 A1 A1c
1c
a
b
A1
C1
1h
Sample ID
Clade ID
Ladder 15P 14P 13P 12P 11P 10P 9P 8P 7P 6P 5P 4P 3P 2P 1P Ladder
C1h C1h A1var1 C1h C1h C1h C1h C1h C1h A1 A1 C1h C1h C1h C1h
HD
a
A1
b
1h
C1

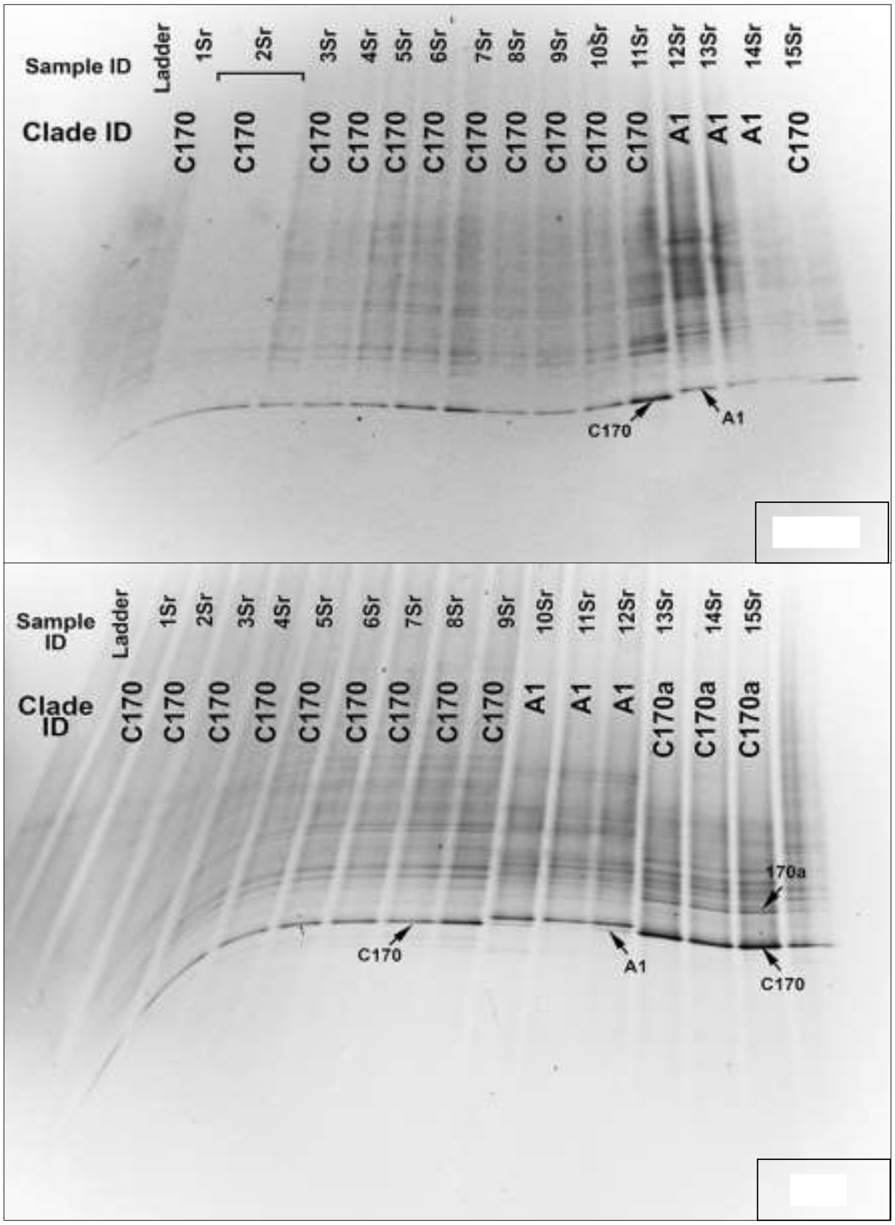

Sample ID
Ladder
1Sr
2Sr
3Sr
4Sr
5Sr
6Sr
7Sr
8Sr
9Sr
10Sr
11Sr
12Sr
13Sr
14Sr
15Sr
Clade ID
C170
C170
C170
C170
C170
C170
C170
C170
C170
C170
C170
A1
A1
A1
C170
C170
A1
Sample ID
Ladder
1Sr
2Sr
3Sr
4Sr
5Sr
6Sr
7Sr
8Sr
9Sr
10Sr
11Sr
12Sr
13Sr
14Sr
15Sr
Clade ID
C170
C170
C170
C170
C170
C170
C170
C170
C170
A1
A1
A1
C170a
C170a
C170a
170a
C170
A1
C170

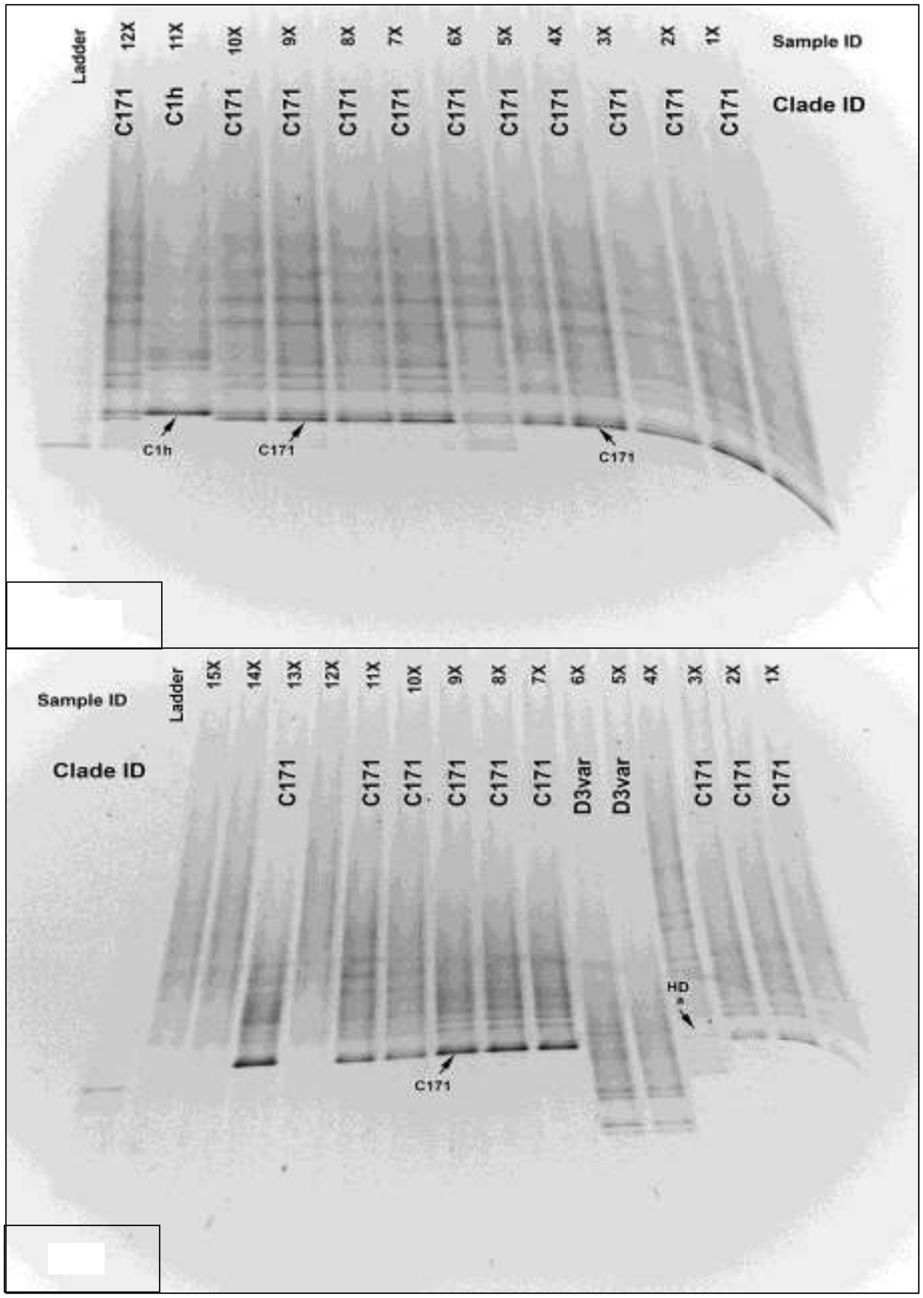

Ladder
12X
11X
10X
9X
8X
7X
6X
5X
4X
3X
2X
1X
Sample ID
C171
C1h
C171
C171
C171
C171
C171
C171
C171
C171
C171
C171
Clade ID
C1h
C171
C171
Sample ID
Ladder
15X
14X
13X
12X
11X
10X
9X
8X
7X
6X
5X
4X
3X
2X
1X
Clade ID
C171
C171
C171
C171
C171
C171
D3var
D3var
C171
C171
C171
HD
C171

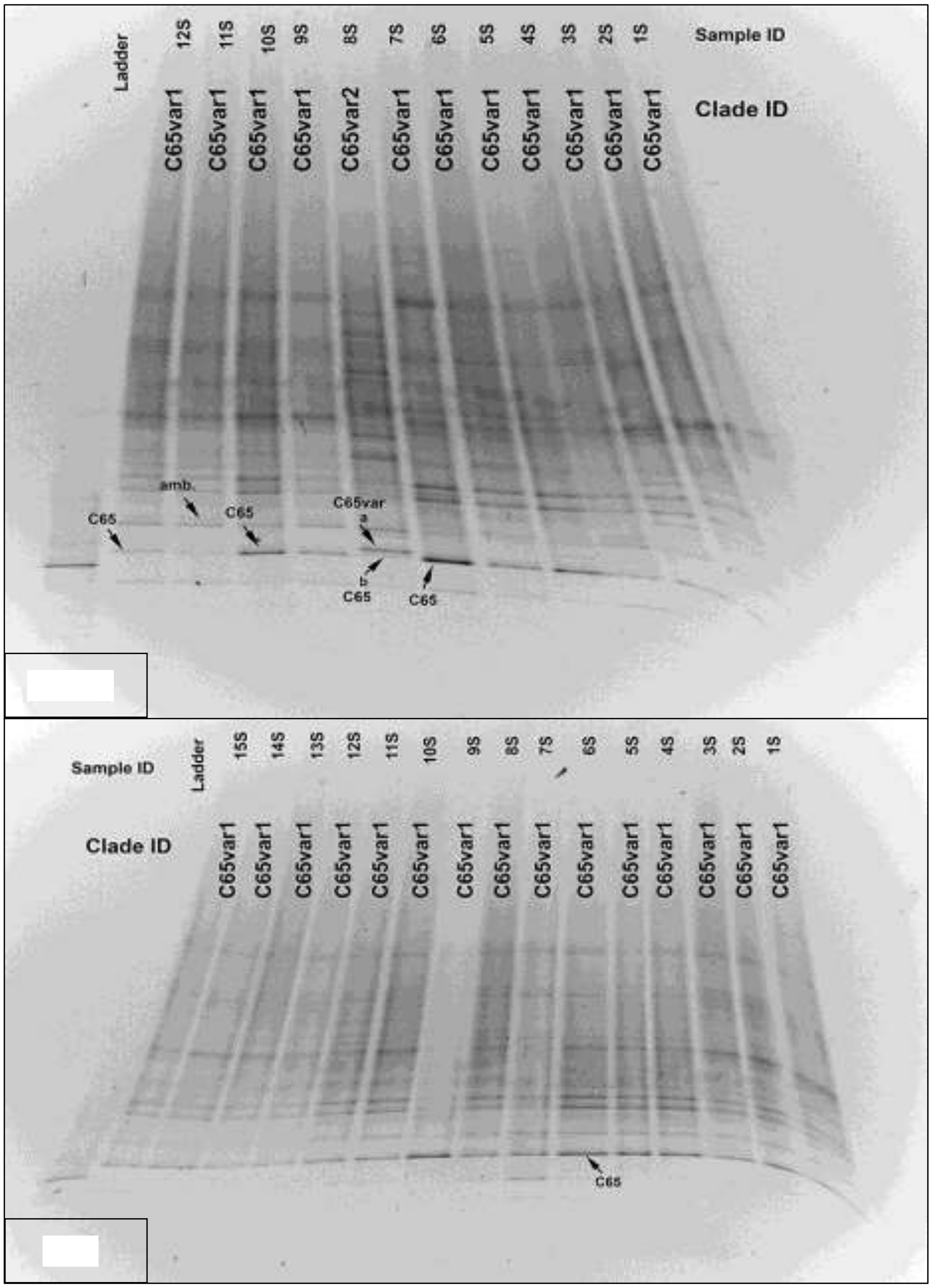

Ladder
12S
11S
10S
9S
8S
7S
6S
5S
4S
3S
2S
1S
Sample ID
C65var1
C65var1
C65var1
C65var1
C65var2
C65var1
C65var1
C65var1
C65var1
C65var1
C65var1
C65var1
Clade ID
C65
amb.
C65
C65var
a
b
C65
C65
Sample ID
Ladder
15S
14S
13S
12S
11S
10S
9S
8S
7S
6S
5S
4S
3S
2S
1S
Clade ID
C65var1
C65var1
C65var1
C65var1
C65var1
C65var1
C65var1
C65var1
C65var1
C65var1
C65var1
C65var1
C65var1
C65var1
C65var1
C65
